# Accelerated stenotic flow in the left anterior descending coronary artery explains the causes of impaired coronary flow reserve: an integrated transthoracic enhanced Doppler study

**DOI:** 10.3389/fcvm.2023.1186983

**Published:** 2023-09-08

**Authors:** Carlo Caiati, Paolo Pollice, Fortunato Iacovelli, Francesca Sturdà, Mario Erminio Lepera

**Affiliations:** Unit of Cardiovascular Diseases, Department of Interdisciplinary Medicine, University of Bari “Aldo Moro”, Bari, Italy

**Keywords:** coronary flow reserve (CFR), accelerated stenotic flow, enhanced transthoracic Doppler echocardiography, critical coronary stenosis, diffuse coronary atherosclerosis, coronary physiology, subcritical coronary stenosis

## Abstract

**Background:**

Accelerated stenotic flow (AsF) in the entire left anterior descending coronary artery (LAD), assessed by transthoracic enhanced color Doppler (E-Doppler TTE), can reveal coronary stenosis (CS) and its severity, enabling a distinction between the microcirculatory and epicardial causes of coronary flow reserve (CFR) impairment.

**Methods:**

Eighty-four consecutive patients with a CFR <2.0 (1.5 ± 0.4), as assessed by E-Doppler TTE, scheduled for coronary angiography (CA) and eventually intracoronary ultrasounds (IVUS), were studied. CFR was calculated by the ratio of peak diastolic flow velocities: during i.v. adenosine (140 mcg/Kg/m) over resting; AsF was calculated as the percentage increase of localized maximal velocity in relation to a reference velocity.

**Results:**

CA showed ≥50% lumen diameter narrowing of the LAD (critical CS) in 68% of patients (57/84) vs. non-critical CS in 32% (27/84). Based on the established CA/IVUS criteria, the non-critical CS subgroup was further subdivided into 2 groups: subcritical/diffuse [16/27 pts (57%)] and no atherosclerosis [11/27 pts (43%)]. CFR was similar in the three groups: 1.4 ± 0.3 in critical CS, 1.5 ± 0.4 in subcritical/diffuse CS, and 1.6 ± 0.4 in no atherosclerosis (*p* = ns). Overall, at least one segment of accelerated stenotic flow in the LAD was found in 73 patients (87%), while in 11 (13%) it was not. The AsF was very predictive of coronary segmental narrowing in both angio subgroups of atherosclerosis but as expected with the usage of different cutoffs. On the basis of the ROC curve, the optimal cutoff was 109% and 16% AsF % increment to successfully distinguish critical from non-critical CS (area under the curve [AUC] = 0.99, *p* < 0.001) and diffuse/subcritical from no CS (AUC = 0.91%, *p* < 0.001). Sensitivity and specificity were 96% and 100% and 82% and 100%, respectively.

**Conclusion:**

E-Doppler TTE is highly feasible and reliable in detecting the CS of any grade of severity, distinguishing epicardial athero from microvascular causes of a severe CFR reduction.

## Introduction

Coronary artery disease (CAD) is the major cause of death in western countries (1). Involvement of the proximal left anterior descending coronary artery (LAD) and the main trunk have the most worrisome consequences and are of maximal clinical relevance to research (2, 3). However, CAD progression can be arrested and even reversed by eliminating the causes of these diseases through appropriate lifestyle changes (4–6). Thus, early detection of CAD is crucially important. It is important to use a non-invasive detection technique that is harmless and therefore repeatable.

Coronary function assessment trumps coronary anatomy evaluation, as recently demonstrated in other studies (7, 8). Absolute coronary flow reserve (CFR) is a seasoned functional assessment for CAD evaluation that can now be obtained non-invasively with transthoracic enhanced Doppler echocardiography (E-Doppler TTE) in the distal LAD (9–14). This distal assessment is more clinically useful than proximal measurements since it takes into account the bleeding off by branches that are proximal to the stenosis and the opposing resistance not only of the stenosis but of global atherosclerosis (athero) proximal to the velocity sampling area (11, 15). Until now, however, CFR with this method has had only marginal clinical relevance since epicardial stenosis assessment, which is essential for the proper interpretation of CFR, can only be done with other techniques (such as invasive and/or x-ray based exams like angiography or coronary computed tomography). These can yield only a morphologic evaluation of the stenosis while completely overlooking the diffuse epicardial athero (16). Thus, the limited application of CFR by E-Doppler TTE is in the setting of microvascular assessment after coronary computed tomography or angiography have morphologically excluded critical disease (but ignoring diffuse athero), which also means there is a possible risk of the overestimation of pure microvascular disease (9).

Accelerated blood flow (AsF) at the stenosis site is a solid functional parameter that aims to maintain flow constant (17). Several invasive attempts at detecting and measuring such AsF have proven to be unsatisfactory for clinical purposes: in the cath lab (18) for scarce feasibility and with transesophageal Doppler echocardiography since it is limited to the proximal portion of the left anterior descending coronary artery (LAD) (19, 20). However, AsF can now be obtained non-invasively and with excellent feasibility (21) in the main trunk and entire LAD using Enhanced transthoracic Doppler echocardiography (E-Doppler TTE) (22, 23). AsF by E-Doppler TTE can thus be used in several clinical applications, including to assess and quantify narrowing of critical (22) and, most importantly, subcritical stenosis (23). Moreover, as preliminarily demonstrated, it can be used to assess diffuse athero (24), and to predict plaque burden as assessed by intracoronary ultrasound (IVUS) (25) and fractional flow reserve (FFR) (26). Such AsF reflects the minimal cross sectional area of the stenosis, the most important determinant of stenosis resistance. This is because dynamically, for any given level of flow, the minimal stenotic area appears as a second order term in both viscous and separation losses (27). For this reason, the hemodynamic burden of epicardial athero, both local or diffuse, can be functionally tracked by the AsF, which might explain the athero impact on the CFR.

A blunted CFR should primarily be explained by the following 3 athero conditions potentially tracked down by AsF: first, the presence of a critical stenosis as assessed by high transtenostic blood flow velocity (22), second, a diffuse subcritical disease (16) showing only a mild localized AsF or uniform high velocity (peak diastolic ≥50 cm/s) not explained by anemia and LV hypertrophy (23, 24) and finally, the absence of epicardial athero, indicated by there being both no localized accelerated stenotic flow and velocity below 50 cm/s (24). In the latter case, microcirculatory dysfunction should explain the low CFR (28).

To verify such a hypothesis, we enrolled 84 consecutive patients scheduled for cath with a blunted CFR by E-Doppler TTE that also had a complete Doppler flow mapping of the LMCA and the whole LAD.

## Methods

### Study groups

Among a total of 400 consecutive patients evaluated with both E-Doppler TTE and coronary angiography/IVUS, we found 84 patients (21%) with CFR <2.0 as assessed by E-Doppler TTE. These patients were consecutive and unselected, thus including those with large body habitus. They underwent E-Doppler TTE for one or more than one of these reasons (inclusion criteria): angina, high risk factors for athero (including previous MI), with follow up of LAD angioplasty. Exclusion criteria were: previous coronary artery bypass graft surgery, and contraindications to adenosine administration (asthma, chronic obstructive pulmonary disease with signs of wheezing, sinus node disease, and advanced atrio-ventricular block) (21). All patients were in a stable setting. Given the exploratory nature of the study, no sample size was formally computed, although it was assumed *a priori* to be 60–70 patients. The study protocol was approved by the Policlinico di Bari, Bari, IT, Institutional Review Board. All patients provided informed consent to take part.

### Research methodology summary

All patients underwent first E-Doppler TTE. Before starting the ultrasound examination the heart rate was lowered below 60 b/m (see below: HR lowering protocol). The E-Doppler TTE examination consisted first of Doppler flow mapping in the LMCA and the whole LAD, then in the distal LAD, blood flow velocity was recorded at the baseline and maximal hyperemia for the assessment of the coronary flow reserve. After that the patients underwent coronary angiography examination (see below: Coronary angiography and IVUS). During coronary angiography, 3 patients also underwent intravascular ultrasounds based on the decision of the Cath Lab Staff.

### Color flow mapping in the LMA and the whole LAD

Ultrasound equipment and technologies (see Supplementary Material: Detailed Methods, which covers this method’s details).

Ultrasound setting (see Supplementary Material: Detailed Methods, which covers this method’s details).

LAD segmentation and anatomy. Color Doppler detection of LAD flow was attempted throughout the LAD by sequentially scanning the proximal, middle, and distal portions (22, 23) (see Supplementary Material: Detailed Methods, which covers this method details about the LAD segmentation and anatomy).

Ultrasound plane orientation. The whole parasternal area was explored from top to bottom using traditional (11) and new approaches, followed by the apical area, as previously described for the B-mode visualization of the LMCA (29) modified for the Doppler recording of blood flow throughout the LAD (see Supplementary Material: Detailed Methods, which covers this method details about the LAD ultrasound plane orientation).

One major advance in plane orientation, apart from the apical views previously described (23), consists of a new parasternal approach for the proximal and mid segments. Briefly, the color flow of the proximal and mid-LAD was substantially improved first by placing the patient in extreme lateral decubitus in order to displace the left lung laterally and uncover the heart as much as possible, and then exploiting the cardiac notch of the left lung by moving the transducer as far as possible laterally on the left hemithorax while maintaining optimal heart insonification. Since the sulcus is much more depressed than the pulmonary conus, this shift of the probe to the left means that the artery is located in the central part of the sector, with maximal ultrasound energy and a narrower theta angle. In this way, a minimal inferior angulation of the probe is more successful in transecting tangentially the depressed content of the sulcus (both proximal and mid part) achieving a narrower theta angle and thus making it possible to record flow for a longer tract in the same plane. Since the probe is very laterally displaced, the diagonal branches are also almost regularly transected and properly Doppler insonified.

#### Heart rate lowering protocol

All patients with a heart rate (HR) of >65 bpm underwent the HR lowering protocol, receiving 0.7 mg oral delorazepam followed by 100 mg oral metoprolol. Thirty minutes later, their HR was checked: if it was ≤60 bpm, they underwent the E-Doppler TTE scan; if it was still >60 bpm, intravenous metoprolol 5 mg was administered over 10 min. In a few cases with persistent HR > 65 b/m, pre-treatment for 3 days with Ivabradine 7.5 mg bis in die before the E-Doppler TTE was prescribed (23).

#### Coronary flow reserve assessment

In our study group, CFR was obtained in the distal LAD by a transthoracic enhanced Doppler study (12, 28). We used a recently validated ultrasound approach that enhances blood flow velocity recording by using a more sensitive Doppler technology based on power Doppler mode (convergent color Doppler). This was associated only in a few cases (10%) with a very poor quality Doppler curve, while the pharmacologically reduced heart rate further improves coronary blood flow signal by reducing clutter artifacts (23). With this new approach, we seldom needed to use contrast enhancement (28). However, in a difficult chest, especially if the heart rate cannot be lowered below 60 b/m, contrast medium remains a major aid. This was required in only 1 patient. Since Levovist® was withdrawn from the market, we used another contrast medium (Sonoview®, Bracco Diagnostics) (30), employing a simplified procedure based on the previously validated use of Levovist® (11). Since after a bolus the duration of enhancement is longer than 3 min, we injected ¼ of the drug vial as a bolus using the same intravenous line as for Adenosine, immediately before starting Adenosine. In this approach, the duration of the enhancement covers the 3 min of infusion of adenosine. After a good signal was attained at baseline [the color flow mapping recording was made in a second harmonic while the color-guided pulsed wave (PW) Doppler was in fundamental mode], Adenosine infusion was started to also enable enhancement coverage of the adenosine step.

#### Plane orientations

As previously described, plane orientation consisted of a modified foreshortened two-chamber view obtained by sliding the transducer superiorly and medially from an apical two-chamber position (11, 12).

#### Adenosine infusion

Intravenous adenosine was infused at the dosage of 140 μg/kg per min over 3 min. The recording of hyperemic flow velocity by PW Doppler was started as soon as the color signal showed an increased velocity (brighter color hue or appearance of aliasing) or, in any case, within 2 min from the beginning of drug administration and continuing until the end of the third minute (31).

#### Atherosclerosis detection protocol

**Figure 1:**
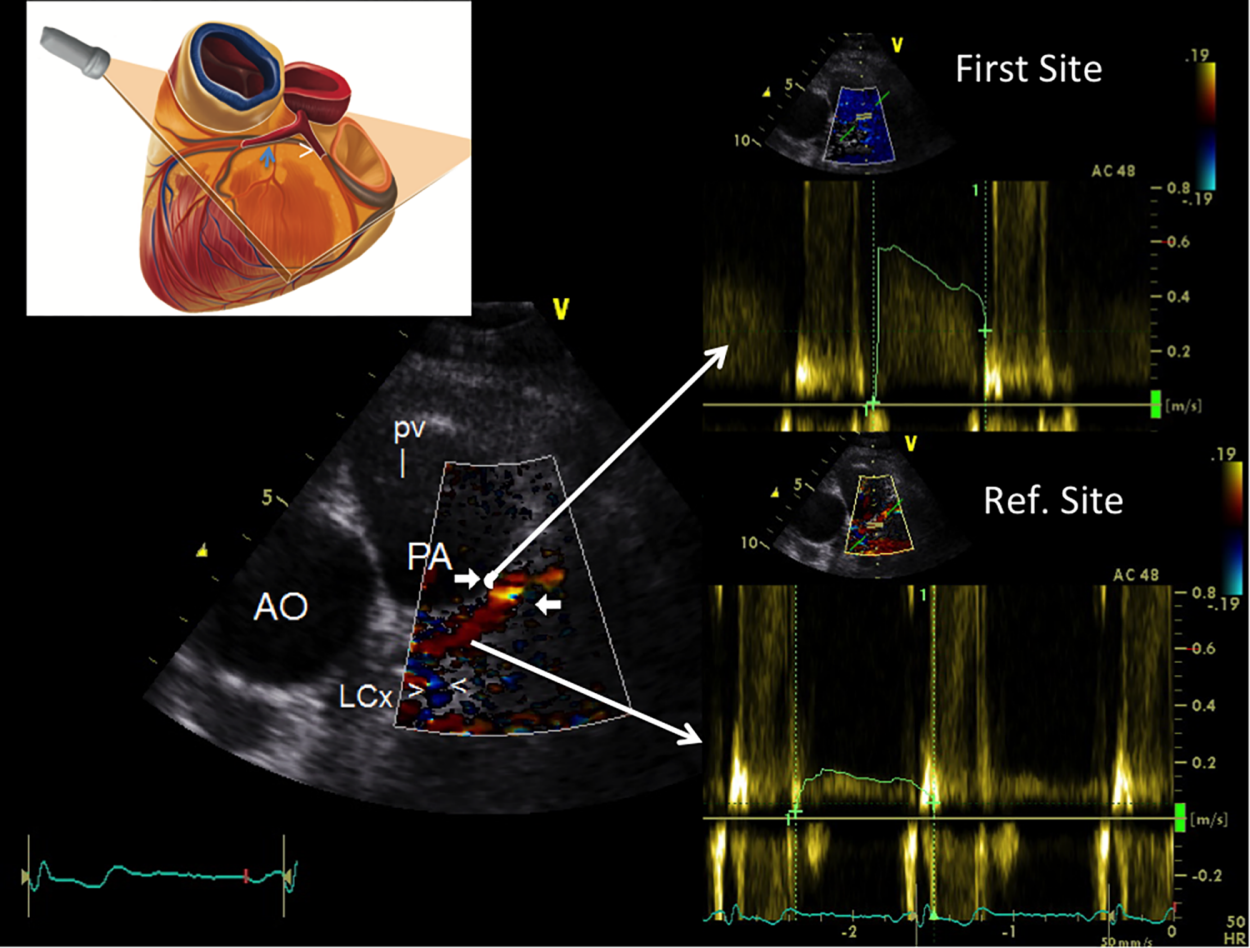
Example of how to perform color-guided PW Doppler sampling in the LAD. Plane orientation is shown on the top left. Color flow is recorded in the proximal LAD (coded in red) along with flow in the proximal LCx [blu signal as it is away from the transducer (arrowheads)]; an area of aliasing (accelerated flow) is shown in the proximal LAD (indicated by arrows); based on color guidance, PW wave Doppler recording is attained at the aliasing area first and then in a more proximal reference portion (reference site); the Doppler curves (on the right side) are then used to calculate %AsF and % CSA reduction. PW, pulsed wave Doppler; LAD, left anterior coronary artery; LCx, left circumflex coronary artery; AsF, accelerated stenotic flow; CSA, cross sectional area.

The protocol applied for the atherosclerosis detection study was: a blinded E-Doppler TTE evaluation performed one day before the scheduled catheterization. The LMCA and LAD were color convergent Doppler- (CCD) mapped, and on color guidance, conventional pulsed-wave Doppler was used to quantify blood flow velocity (BFV) in the LMCA, and in the proximal, mid, and distal LAD. A schematic example that illustrates the methodology used for flow mapping is reported in Figure 1 and such a methodology has been previously described in detail (22). Briefly, each of the 3 LAD segments defined above were systematically mapped during diastole with the coronary still and stable in that part of the cardiac cycle (32), which is much more easily done when the diastole is lengthened as when the heart rate is below 60 b/m (21). If aliasing was evident in LAD color flow mapping, we first sampled (first site) the portion in which the color Doppler signal appeared aliased (Figure 1). If aliasing was not evident, we sampled the whole visualized segment to obtain the fastest velocity possible. Second (second site or reference site), we recorded by sampling proximally (Figure 1) or distally to the area with the highest recorded velocity, making sure that the color did not seem to be disturbed. The mean angle of incidence between the Doppler beam and the direction of the blood flow (the *θ* angle) was small and always corrected.

All the patients with a HR of >60 bpm underwent the HR lowering protocol.

#### Echocardiographic data analysis

All the Doppler readings were performed by the same operator (CC) blinded to the catheterization results (angiography), as all the E-Doppler TTE studies were performed one day before the catheterization. In addition, the maximum recorded length of the LAD color Doppler flow signal in the proximal mid and distal segment was quantitatively measured using calipers (19).

#### E-Doppler TTE: pulsed-wave Doppler analysis

The peak and time velocity integrals of the diastolic waves were measured at two sites in the LMCA and in each LAD segment. The AsF was calculated as the percentage difference between the peak velocity intervals (diastolic peak at first site—diastolic peak at second site/diastolic peak at second site) *100 (Figure 1) (19, 22). The variability and intra and inter-observer reproducibility between these two measurements have been previously reported (12, 23).

#### Reproducibility

Inter-observer Doppler parameter reproducibility was assessed. The inter-observer test was assessed by performing the exam twice 4 days apart by two operators (CC and PP) in a randomly selected group of 14 consecutive patients (9 stenosis, 7 in the proximal, 2 in the mid LAD). The % increment of velocity (AsF) and % CSA stenosis were twice determined. In addition, the LAD global color length, number of LAD segments with aliased color signal, LAD site of each aliased color signal, and theta angle correction before color-guided PW Doppler recording were also attained twice.

#### E-Doppler TTE vs. quantitative coronary angiography: Doppler determination of percentage area stenosis

The severity of LAD stenosis was assessed using the continuity equation (20, 22). Intra- and inter-observers Doppler parameters reproducibility has been also preliminary reported (23). (see Supplementary Material: Detailed Methods, which covers this method in detail).

### Coronary angiography and IVUS

All the patients underwent coronary angiography through the trans-femoral or trans-radial route, depending on the physician's judgment and the patient's anatomy and clinical condition.

All angiographic studies were interpreted blind, since they were performed as routine studies. The coronary stenosis was visually assessed based on multiple projections by one investigator, unaware of the TTE Doppler results. The presence of minimal luminal irregularities was specifically checked.

### Quantitative coronary angiography

In 47 stenoses from 39 patients in the study group, the maximum (reference) proximal and distal luminal diameters, as well as the minimum luminal diameter itself were assessed from digital images using an off-line computerized bidimensional quantitative coronary angiography (QCA) analysis system (QAngio 7.3, Medis medical imaging systems bv, Leiden, The Netherlands). According to the last consensus document published (33), after having chosen the best projection to avoid branch crossing or foreshortening of the lesion to analyze, a calibration was conducted using the catheter size (6 French) in order to determine the actual caliper of the coronary artery lumen. The diameter measurements were used to calculate the minimum and reference luminal cross-sectional area (assuming a circular cross-section), after which the percentage cross-sectional area was automatically calculated as:

$$\text{\%}A_{s}=(A_{\mathrm{ref}}-A_{s})/A_{ref}\times 100$$

### Interpretation of the coronary angiogram in patients without critical LAD stenosis

After catheterization, the patients were subdivided into two groups based on the extent of atherosclerosis on the diagnostic coronary angiograms (34). Classifications were made by consensus by three investigators blinded to the study results. The LAD was classified as smooth (group 1) or irregular (group 2).

Group 1 included patients with angiographically smooth LAD segments (with a particular focus on the proximal LAD segment) and neither stenosis nor clear parietal irregularities of the right and circumflex coronary artery.

Group 2 included patients with mild/diffuse atherosclerosis. Patients with LAD luminal irregularities (with particular focus on the proximal LAD segment) (34) with or without stenosis <50% and some degree of stenosis (either > or ≤50%) in the right or circumflex coronary artery on diagnostic angiography were considered to have LAD early atherosclerosis. In cases of IVUS (performed in 3 patients) a plaque burden >60% in the proximal and mid-LAD segments was considered diffuse athero (35).

*IVUS procedure and analysis of IVUS data* (see Supplementary Material: Detailed Methods, which covers this method in detail) (36).

Before the diagnostic catheterization procedure, written informed consent was obtained from all patients in accordance with guidelines established by the Committee for the Protection of Human Subjects at Brigham and Women's Hospital.

### Statistical analysis

Continuous variables are expressed as mean values ± 1 SD. A *p*-value <0.05 was considered significant. A one-way between-groups analysis of variance was conducted to explore the difference in CFR in the 3 athero subgroups and the Tukey HSD test was used for *post hoc* comparison. Pearson product-moment correlation coefficient was used to investigate the relationship between CFR and AsF. An independent-samples *t*-test was conducted to compare AsF in the different athero subgroups. Categorical variables were counted with percentages, and compared using a Chi-squared or Fischer`s exact test, depending on group sizes. The best cut-off point for AsF was empirically estimated using receiver operating characteristic (ROC) curves. Bootstrapping (5,000 resampling) was used for a 95% confidence interval assessment of the cutoff. The agreement between QCA and E-Doppler TTE in terms of the percent cross-sectional area (CSA) of the stenosis was evaluated using linear regression analysis and the Bland-Altman method (37). Test–retest assessments were performed by K statistics and Intra-class correlation coefficient and also by the Bland–Altman method, which allows determination of the coefficient of repeatability that is calculated as 1.96 (or 2) times the standard deviation of the differences between the two measurements (37). Statistical calculations were performed using IBM SPSS statistics version 23, Armonk, NY: IBM Corp, and MedCalc Statistical Software version 19.1.3 (MedCalc Software bv, Ostend, Belgium).

## Results

### Coronary angiography/IVUS

**Figure 2:**
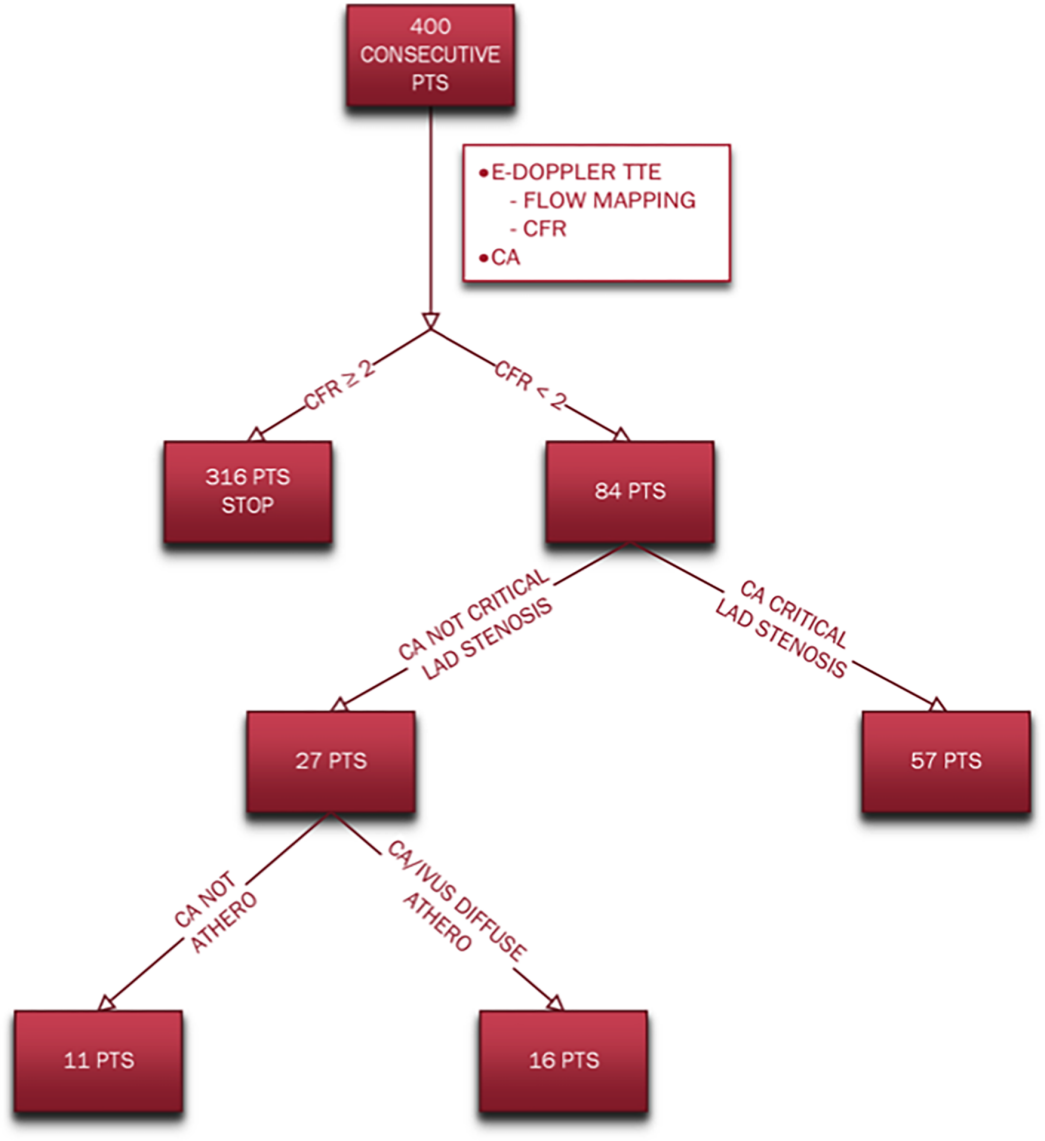
Flow chart indicating the different subgroups of coronary atherosclerosis based on angiography/IVUS and CFR results. CA, coronary angiography; CFR, coronary flow reserve; E-Doppler TTE, enhanced transthoracic Doppler; IVUS, intravascular ultrasounds; pts, patients.

Different subgroups of coronary atherosclerosis were obtained based on coronary angiography/IVUS and CFR results are summarized in the flow chart (Figure 2).

The coronary angiography showed that most of the patients (57, 68%) had either 1 or more than 1 critical localized LAD stenosis while 27 (32%) did not. Among these last 27 patients with no LAD critical stenosis, only the minority [11 patients (13% of the whole group)] showed no signs of CAD involvement of the LAD and the other 2 coronaries; instead, the majority of this subgroup (16 patients [19% of the whole group] had angiographic signs of a diffuse atherosclerosis (lumen irregularities) and sometimes added mild segmental LAD CAD (confirmed in 3 pts also by IVUS: diffuse plaque with max plaque burden >60%) and always of either critical or subcritical right and/or circumflex coronary arteries stenosis as well.

The demographic and clinical findings broken down into the 3 angiographic/IVUS subgroups (no stenosis, mild/diffuse, and critical stenosis) are reported in Table 1.

**Table 1:** Demographic, clinical, and echocardiographic data in the three angiographic/IVUS subgroups.

* *		No athero (11 patients)	Critical stenotic athero (57 patients)	Diffuse subcritical athero (16 patients)
Age (years)		72 ± 9	68 ± 9	69 ± 11
BMI		24 ± 3	27 ± 3	28 ± 8
Sex	Male, *n* (%)	4 (36)	48 (86)	10 (67)
	Female, *n* (%)	7 (63)	8 (14)*	5 (33)
Angina				
Typical	*n* (%)	5 (45)	16 (43)	6 (40)
Atypical	*n* (%)	3 (27)	7 (19)	1 (7)
Diabetes	*n* (%)	2 (18)	23 (41)	5 (31)
Previous CAD	*n* (%)	2 (18)	34 (61)*	5 (33)
Previous MI	*n* (%)	0 (0)	16 (28)	5 (33)
Previous PTCA	*n* (%)	0 (0)	13 (23)	1 (7)
Hypertension	*n* (%)	7 (64)	50 (88)†	10 (62)
DCM	*n* (%)	3 (27)	6 (12)	6 (40)‡
Tobacco Smoke	*n* (%)	2 (18)	9 (16)	4 (25)
#cig per day		3 ± 6	3 ± 10	5 ± 11
Total cholesterol mmol/L (mg/dl)		4.54 ± 1.18 (175 ± 45)	4.67 ± 1.29 (180 ± 50)	4.64 ± 1.0 (179 ± 38)
HDL mmol/L (mg/dl)		1.34 ± 0.3 (52 ± 11)	1.13 ± 0.3 (44 ± 12)	1.18 ± 0.41 (46 ± 16)
LDL mmol/L (mg/dl)		2.63 ± 1.06 (102 ± 41)	2.84 ± 1.04 (110 ± 40)	2.88 ± 0.82 (111 ± 31)
Triglycerides mmol/L (mg/dl)		2.81 ± 1.24 (108 ± 14)	3.59 ± 2.87 (138 ± 111)	2.83 ± 1.05 (109 ± 41)
LVEDD, mm		47 ± 14	49 ± 6	54 ± 9
LVESD, mm		35 ± 13	35 ± 10	43 ± 13
LV EF (%)		49 ± 14	52 ± 15	47 ± 15
QCA % CSA (%)		–	87 ± 11	47 ± 6**
QCA Minimal DS (mm)		–	0.9 ± 0.5	1.7 ± 0.3**

IVUS, intracoronary ultrasound; BMI, body mass index; CAD, coronary artery disease; MI, myocardial infarction; PTCA, percutaneous coronary angioplasty; DCM, dilated cardiomyopathy; HDL, high density lipoproteins; LDL, low density lipoproteins; cig, cigarettes; LVEDD, left ventricle end diastolic dimension (long axis parasternal view); LVESD, left ventricle end systolic dimension (long axis parasternal view); EF, ejection fraction. QCA, quantitative coronary angiography; CSA, cross sectional area; DS, diameter stenosis.

* *p* < 0.01 vs. no athero group.

** *p* < 0.01 vs critical athero.

† *p* < 0.05 vs. subcritical athero.

‡ *p* = 0.051 vs. critical stenotic athero group; QCA only in 5 patients with subcritical athero.

### CFR assessment

Blood flow velocity recording in baseline conditions and during adenosine infusion was attempted first in the distal LAD. If no or an unsatisfactory recording was obtained in the distal segment, a recording was attempted in the mid-portion (2 pts).

**Figure 3:**
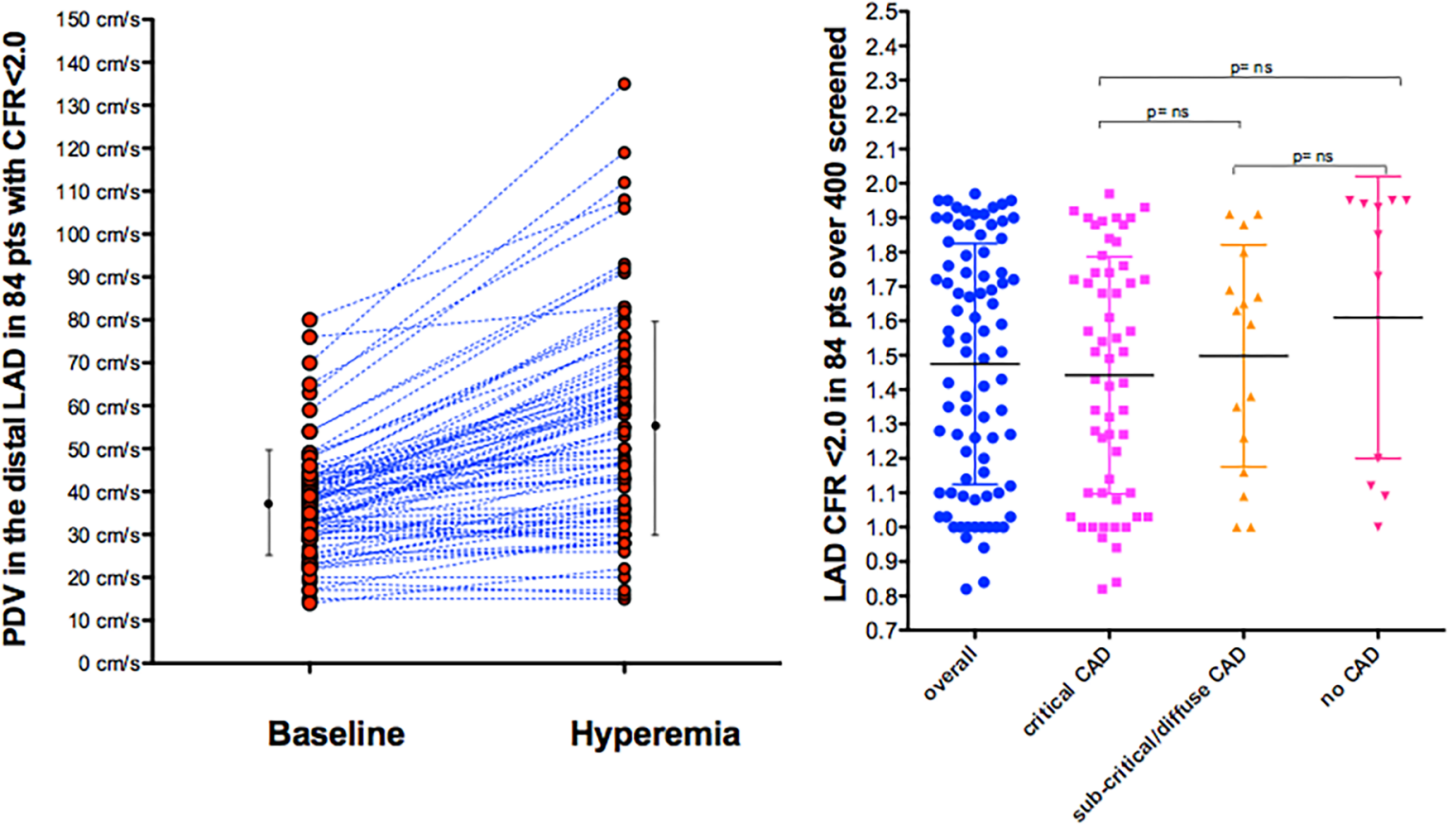
Velocities and CFR as assessed by E-Doppler TTE. Left Panel: Scattergram of velocities in the distal LAD before and after adenosine (mean and SD are also shown) in the whole group; Right Panel: individual value bar graph of CFR overall and also broken down in the 3 subgroups of LAD athero (mean and SD are also reported in each subgroup). A one-way between-groups analysis of variance and Tukey HSD for *post hoc* comparison were used; PDV, peak diastolic velocity; LAD, left anterior descending coronary artery; CFR, coronary flow reserve; CAD, coronary artery disease.

E-Doppler TTE, along with lowering HR, greatly enhanced the success rate in recording adequate PW Doppler signal from the LAD (21). In our global study group of 84 consecutive patients with a CFR <2.0, the baseline mean and peak diastolic velocity was 28 ± 9 cm/s and 37 ± 12 cm/s, respectively, and during hyperemia 43 ± 20 and 56 ± 24 cm/s, showing a CFR for mean and peak at 1.5 ± 0.3 and 1.5 ± 0.4, respectively (Figure 3).

CFR was not different among the 3 angiographic grades of athero severity, which were 1.4 ± 0.3 in the critical CAD subgroup, 1.5 ± 0.4 in subcritical/diffuse CAD, and 1.6 ± 0.4 in no CAD (F = 1.16, *p* = ns) (Figure 3).

#### Adenosine infusion

No major adverse reactions occurred after adenosine infusion. Twenty patients complained of hyperpnoea that tended to abate by the end of the infusion.

A slight modification of heart rate was observed after adenosine. Heart rate increased from 64 ± 10 to 74 ± 13 bpm (p < 0.05). Arterial pressure did not show a significant change after adenosine (130 ± 17 mmHg systolic and 72 ± 11 mmHg diastolic at rest and 131 ± 18 mmHg systolic and 72 ± 12 mmHg diastolic during adenosine [*p* = ns]).

### CFR and accelerated stenotic flow: overall direct comparison

Flow velocities in the entire LAD were obtained for all 84 patients (max length of LAD color flow was 73 ± 17 mm).

**Figure 4:**
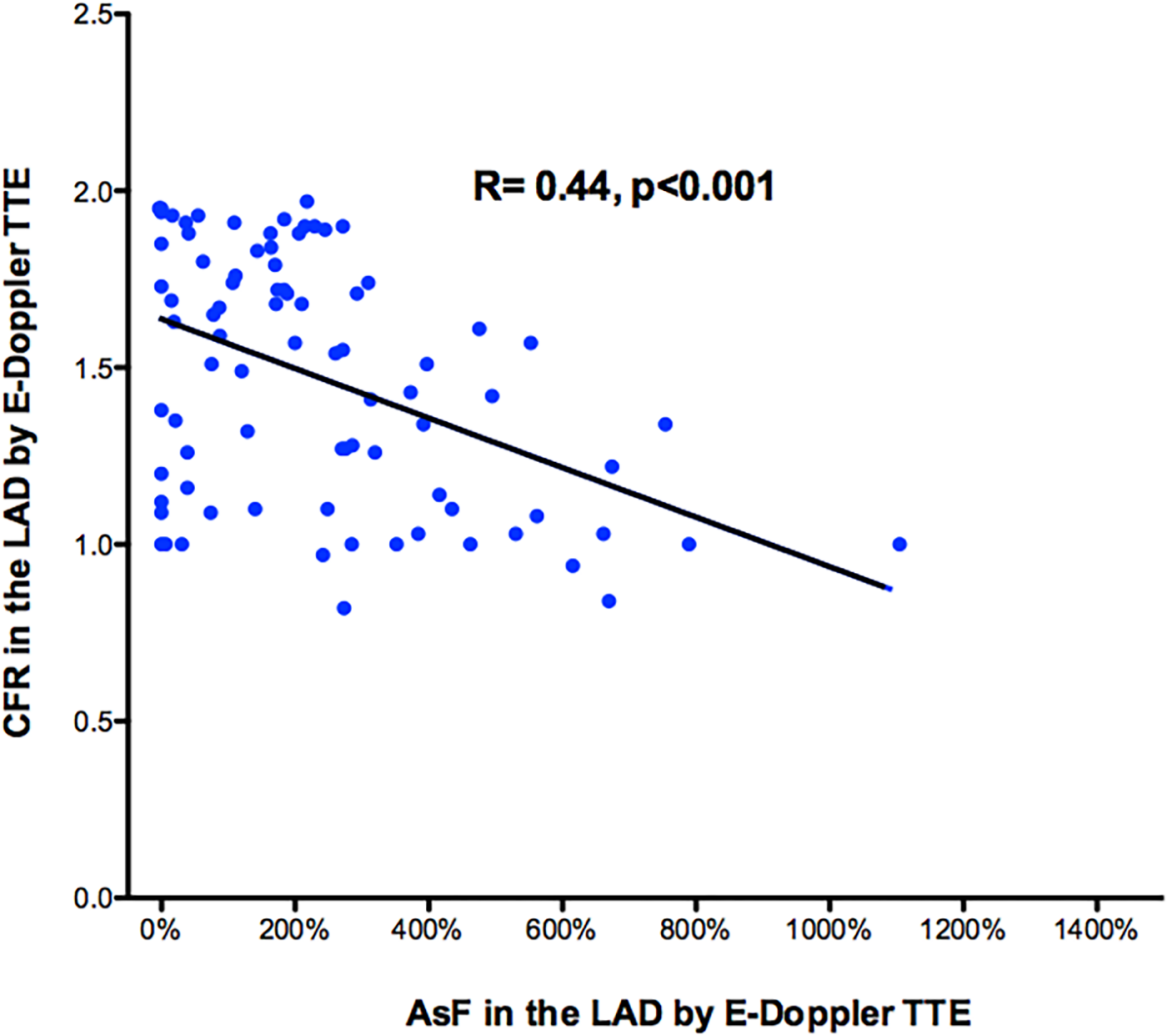
Scattergram showing the relation between percentage CFR and AsF in the LAD for the whole 84 patient group. It shows a moderately strong negative correlation between the max accelerated stenotic flow expressed as the percentage increment of velocity (*x*-axis) and distal CFR (*y*-axis). Pearson product-moment correlation coefficient was used. Line of correlation (continuous line) is drawn. CFR, coronary flow reserve; AsF, accelerated stenotic flow; LAD, left anterior descending coronary artery.

Overall, at least one segment of accelerated stenotic flow in the LAD was found in 73 patients, while in 11 it was not. A higher segmental acceleration predicted a more blunted CFR. There was a moderately strong negative correlation between the max accelerated stenotic flow expressed as the percentage increment of velocity and the CFR, *r* = −.44, *p* < 0.001, with high levels of % velocity increment associated with lower levels of CFR (Figure 4).

### Localized acceleration of stenotic flow in the LAD in predicting critical angiographic stenosis

**Figure 5:**
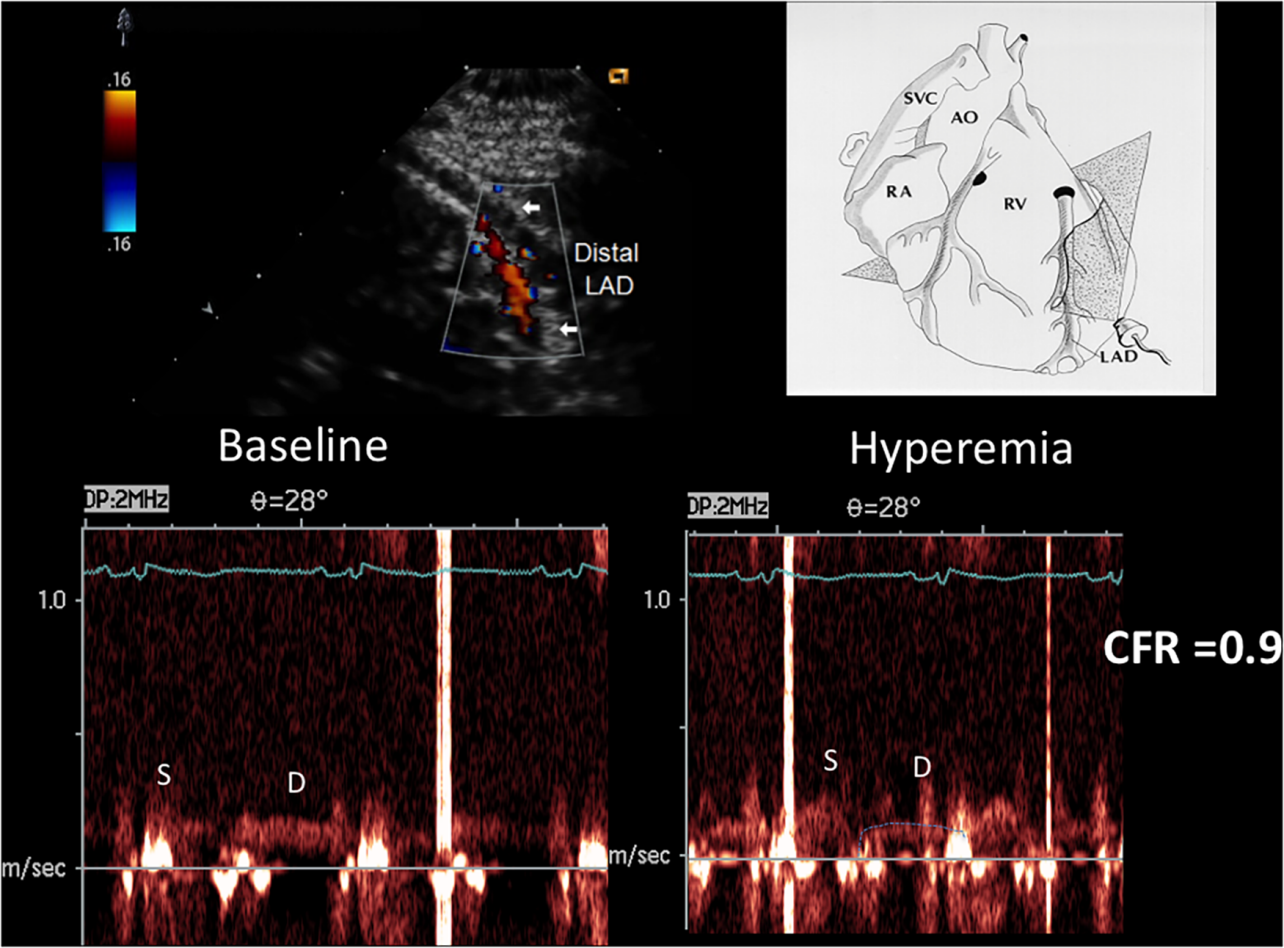
CFR in the distal LAD by E-Doppler TTE in a patient with a critical LAD stenosis as assessed by angio. At the top, color flow in the distal LAD (in red); on the right, a cartoon of the tomographic plane orientation to obtain the LAD insonification. At the bottom, pulsed Doppler spectral tracing of the blood flow velocity in the distal LAD at baseline (left) and at maximal Adenosine-induced hyperemia (right); note the prevalent diastolic BF velocity at the baseline with a minimal predominance of the diastolic flow; during hyperemia the S/D ratio gets inverted and at the same time the diastolic flow gets reduced indicating a possible stealing phenomenon. The CFR (peak hyperemic diastolic velocity/peak resting diastolic velocity) is thus below 1 (0.9). S/D ratio, ratio of the systolic to diastolic waves of coronary flow velocity; CFR, coronary flow reserve; LAD, left anterior descending coronary artery.

**Figure 6:**
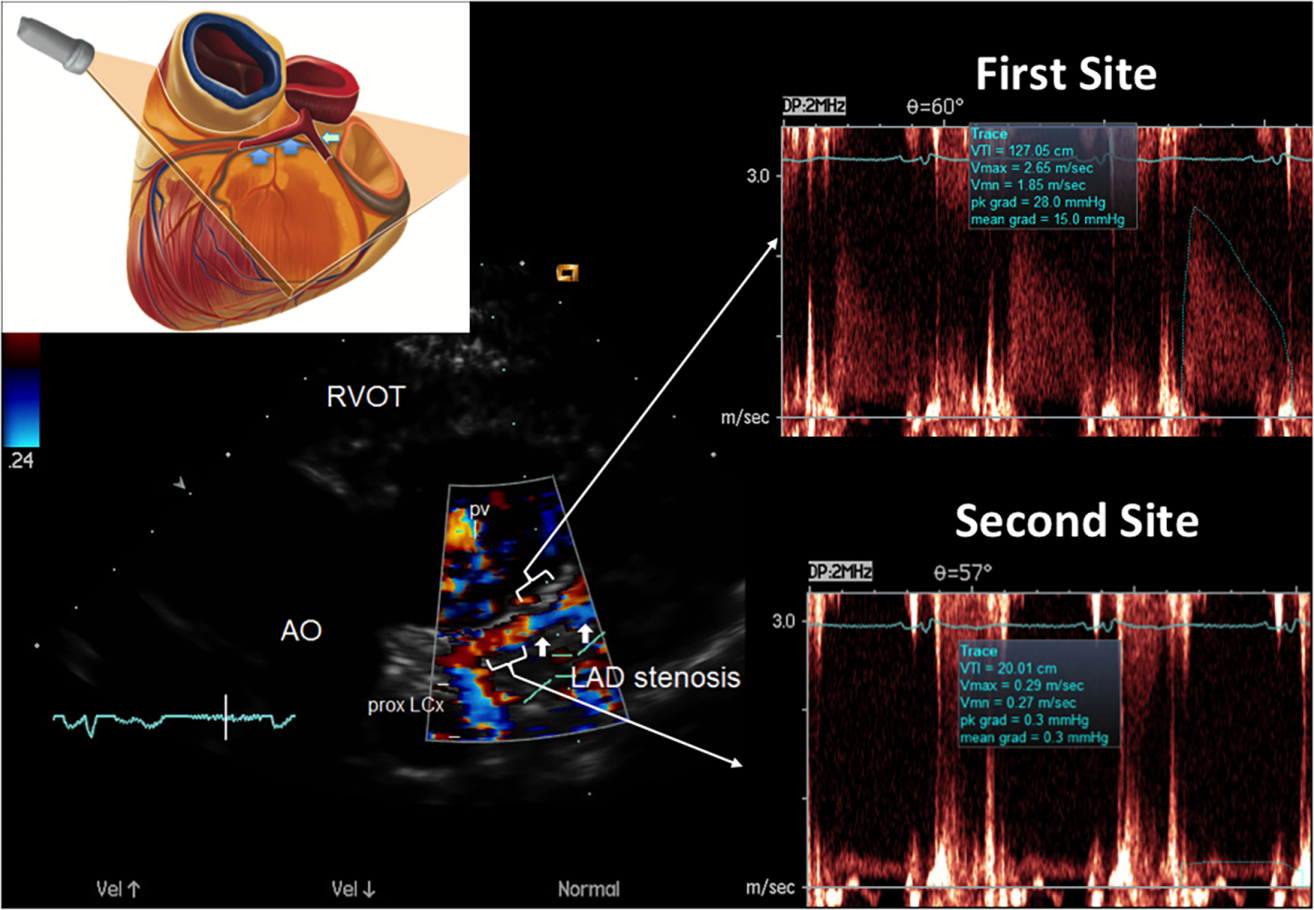
E-Doppler TTE AsF tracking in the LAD in the same patient as that with CFR < 1. Left: long area of aliasing (>1 cm) in the LAD (indicated by arrows) almost 1 cm from the LMCA bifurcation. At the upper left a cartoon that explains the plane orientation to transect the left coronary fossa with its content. In the right area: pulsed-wave Doppler tracing at the aliasing (upper trace) shows much greater BFV than at the distal reference site (bottom trace) so detecting a very high AsF (670% increment of velocity). By applying the corrected continuity equation that utilizes the VTI (time velocity integral) of the 2 curves (first site and second site) the percentage reduction of the area at the stenosis site is 92% indicating a severe stenosis. A proximal LCx color flow is also visualized (the signal is blue because is away from the transducer) LCX, left circumflex coronary artery color flow; RVOT, right ventricular outflow tract; AO, aorta; PV, pulmonary valve; AsF, accelerated stenotic flow.

**Figure 7:**
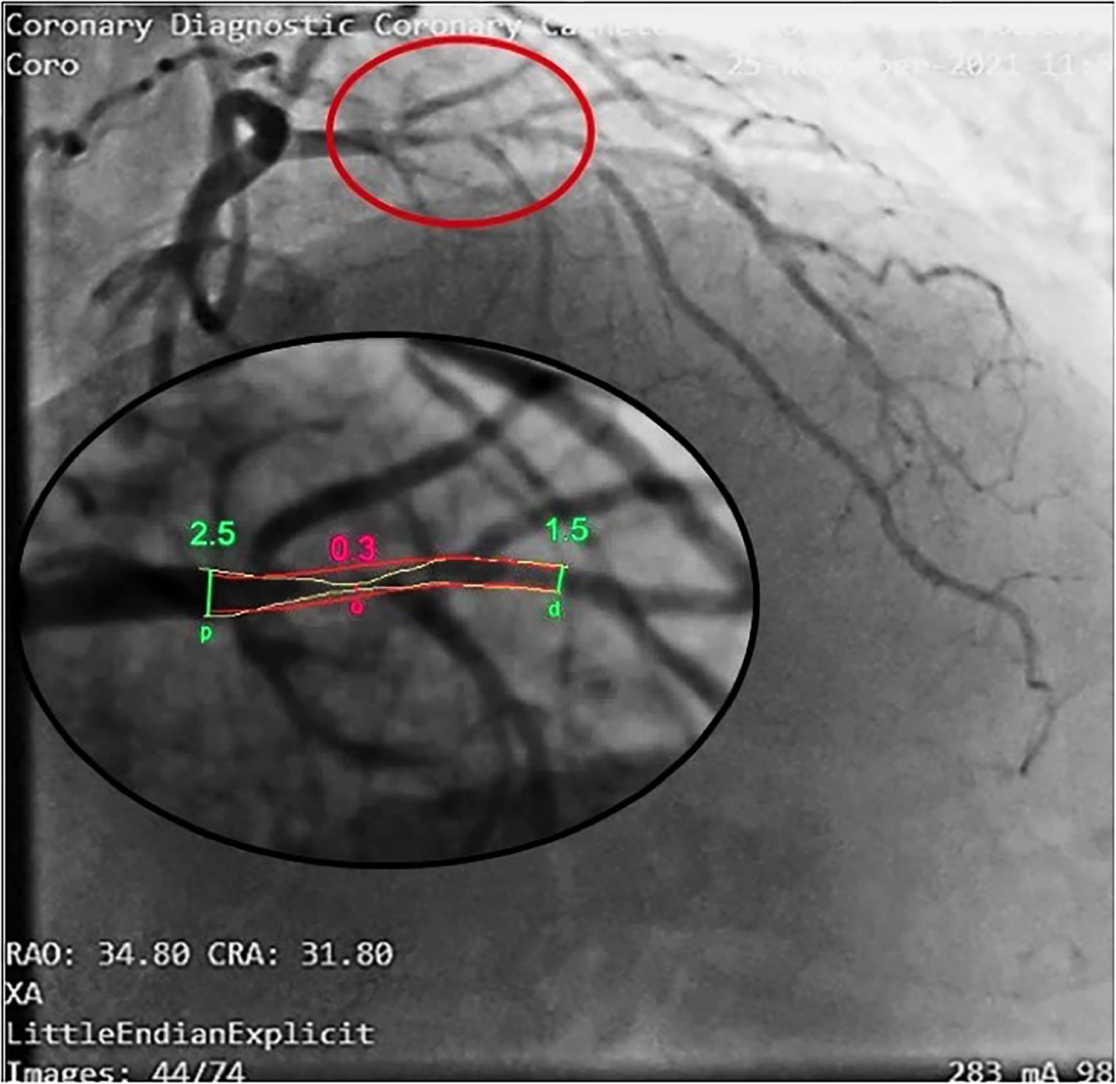
Coronary angiography in the same patient as in Figures 5, 6. In this right oblique view, the site of the stenosis (encircled in red) has been magnified (at the bottom) to measure the minimal and maximal diameters (QCA). Coronary angiography confirms a very tight stenosi almost occlusive of the proximal LAD (QCA = 98% area stenosis) confirming the E-Doppler TTE evaluation in terms of stenosis detection, location, and severity assessment. QCA, quantitative coronary angiography. LAD, left anterior descending coronary artery.

The accelerated stenotic flow helped to detect, locate and assess the severity of the critical angiographic stenosis. Figures 5–7 illustrate this point in a patient with flat CFR and severe proximal LAD stenosis.

Regarding detection, first-site BFV in the LAD was higher in the 57 patients with angiographic evidence of critical disease than in the 27 patients with no critical disease (154 ± 62 vs. 51 ± 16 cm/s; mean difference 102 cm/s, 95% CI: 85–120, *p* < 0.001), whereas velocity at the reference sites was similar (38 ± 10 cm/s vs. 42 ± 12 cm/s; *p* = ns). Consequently, the percentage increase was greater in the former (326 ± 209% vs. 22 ± 31%; mean difference = 304%, 95% CI: 247–360, *p* < 0.001), who also more frequently showed at least one localized aliased color signal: 57 patients (100%) vs. 17 patients (63%) (*p* < 0.05) (see Supplementary Video S1, which shows the movie version of the example reported in Figure 6).

**Figure 8:**
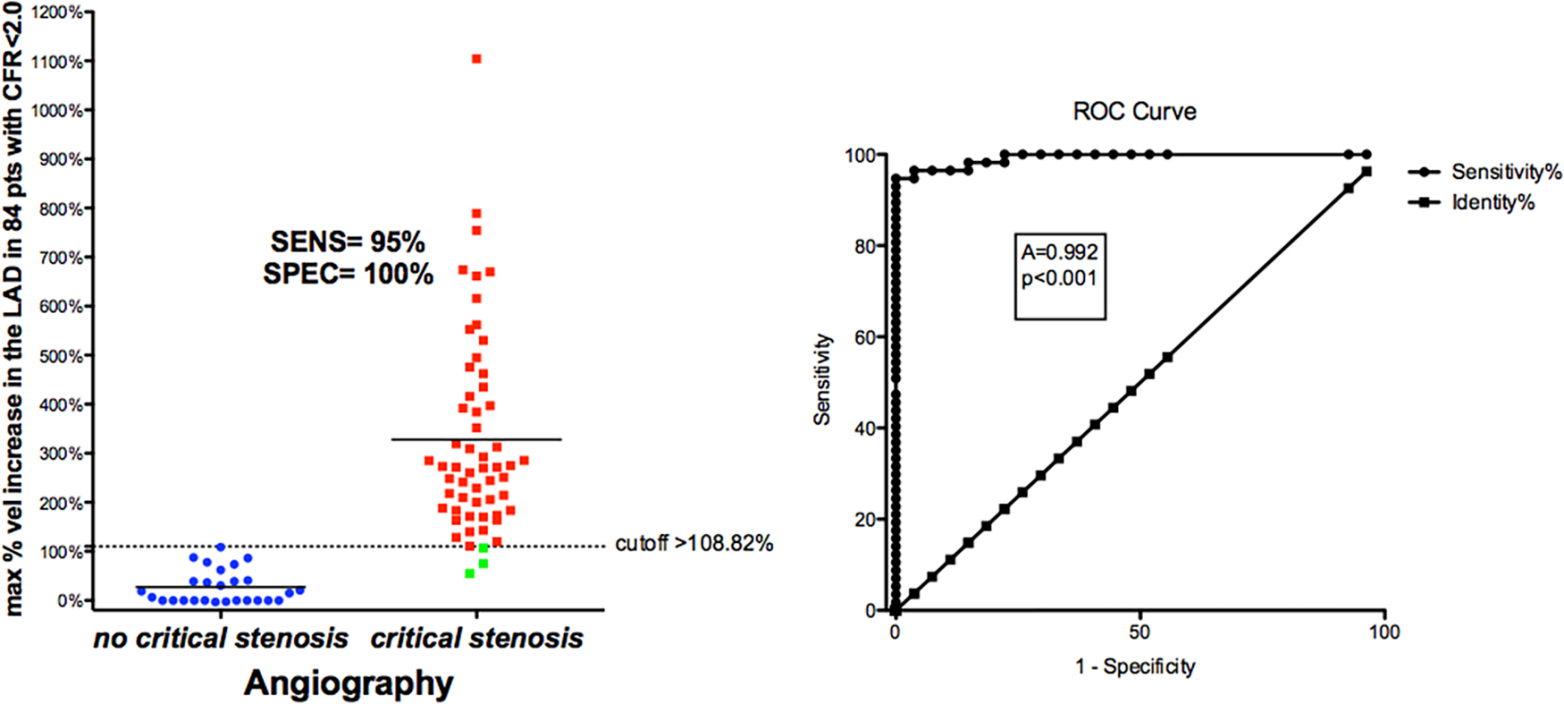
ROC curves in critical CAD. Left: individual value bar graph showing the maximum individual percentage increases in velocity in the LAD segments in patients with and without critical stenosis, and the cut-off value (dashed horizontal line). Right: plot of sensitivity against 1-specificity of the percentage increase in velocity (ROC curves) predicting critical stenosis in the left anterior descending coronary artery, as assessed by angiography. A, area under the curve; vel, velocity; CFR, coronary flow reserve; sen, sensitivity; spe, specificity.

BFV % increase using a cut-off value >109% (bootstrapped 95% CI >87.5 to >108.82) as obtained by ROC curve analysis (area under the curve = 0.99, 95% CI: 0.94–1.0, *p* < 0.0001) provided clinically valuable metrics of the reliability of the E-Doppler TTE in detecting a critical stenosis (Figure 8): sensitivity, specificity, positive predictive value, negative predictive and accuracy value were 96% (95% CI: 85%–99%, 54/57 pts), 100% (95% CI: 87%–100%, 27/27 pts), 100% (54/54 pts), 90% (95% CI: 75%–96%, 27/30 pts), and 95% (95% CI: 90%–99%), respectively.

The 3 false negatives (Figure 8, green squares) were not completely negative (no BFV acceleration at all). In one patient, the AsF in the mid LAD (107%) was just minimally below the cutoff, corresponded to a borderline critical mid LAD stenosis (50%); in the second patient, we mapped the initial part of the mid LAD and a large diagonal that was misinterpreted as the continuation of the main LAD trunk, but we missed the true main trunk that, after the diagonal, had severe stenosis, since the LAD after the diagonal was abruptly bent downward. However, we caught a mild more proximal sentinel stenosis with AsF = 54%. The diagonals can generally be recognized since they are directed leftward while the LAD is directed rightward. The third patient had a mid LAD occlusion that was not properly identified and the distal CFR was measured in a large diagonal reaching to the apex.

Regarding plaque location*,* the location of critical BFV acceleration(s) (color Doppler aliasing zone) strictly corresponded to the critical angio-detected plaque location in the proximal mid and distal LAD (*p* < 0.001, Cramer's *V* = 0.55) (Table 2). However, the Doppler tended to detect less multiple LAD stenosis and to misplace mid-angiographic stenosis as proximal (Table 2). This latter point is related to different criteria in defining the distal end of the proximal LAD segment by Doppler (at the level of the pulmonary valve) or by angio (at the stemming of either the first septal perforator or the first diagonal).

**Table 2:** Cross-tabulation of LAD critical plaque location by E Doppler TTE versus angiography in 84 patients.

		Location of E-Doppler TTE-revealed critical stenosis (*)								
		Prox	Mid	Dist	Prox-mid	Mid-dist	Prox-dist	Prox-mid-dist	No crit sten	Tot Dop
Location of Angio-revealed critical stenosis	Prox	**7**	1	0	1	0	1	0	2	12
	Mid	5	**12**	0	1	0	0	0	0	18
	Dist	1	1	**1**	0	0	0	0	0	3
	Prox-mid	10	4	0	**0**	0	0	0	0	14
	Mid-dist	2	1	1	0	**1**	0	0	0	5
	Prox-dist	1	0	0	0	0	**0**	0	0	1
	Prox-mid-dist	0	0	0	0	1	0	**2**	1	4
	No crit sten	0	0	0	0	0	0	0	**27**	27
Tot angio		26	19	2	2	2	1	2	30	84

LAD, left anterior descending coronary artery; E Doppler TTE, transthoracic enhanced Doppler echocardiography; Prox, proximal; Dist, distal; #patients, numbers of patients; sten, stenosis; Tot Angio, total numbers of patients with critical stenosis in specific LAD location by angiography; Tot Dop, total numbers of patients in specific LAD location by E-Doppler TTE; numbers in bold indicate identity between E-Doppler TTE and Angio.

* *p* < 0.001 vs. angiography.

**Figure 9:**
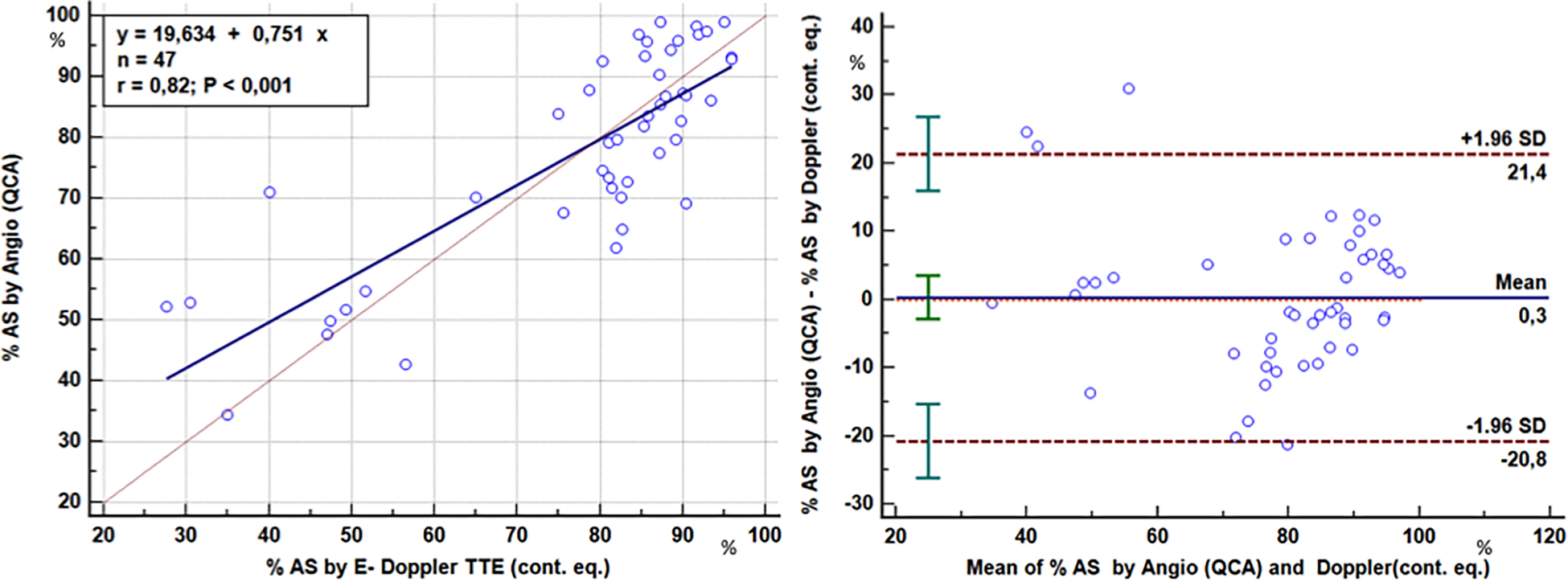
Comparison of the severity assessment of the LAD stenoses: E-Doppler TTE vs QCA. Left panel: Scattergram showing the relation between percentage cross-sectional area reduction at the stenosis site (AS) obtained with two methods: enhanced transthoracic Doppler by the application of continuity equation (E-Doppler TTE, *x*-axis) and quantitative coronary angiography (QCA, *y*-axis). Lines of equality (dotted line) and correlation (continuous line) are drawn. Right panel: Plot of the average value against the difference of the QCA and E- Doppler TTE calculated %AS. The dotted lines represent the boundaries of the mean + 2 SD. Pearson product-moment correlation coefficient and Blandt Altmann plot were used. %AS, percent area stenosis; LAD, left anterior descending coronary artery; cont. eq., continuity equation.

As to stenosis severity, there was an excellent correlation between the Doppler-derived (continuity equation using either the corrected or the non-corrected formula in accordance with the presence or absence of AsF ≥ or <109% respectively) and angiographic QCA % CSA of the LAD stenoses (77 ± 18% vs. 77 ± 16%; *r* = 0.82, *p* < 0.001) in 47 stenoses (critical or subcritical) of 39 patients of the study group (Figure 9). Agreement analysis showed that the maximum difference between the two methods was ∼20% with no significant bias (0.3%) (Figure 9).

#### Acceleration of stenotic flow in the LAD in predicting subcritical/diffuse LAD disease

In the subgroup with no angiographic critical stenosis (27 patients), we found that some patients either had acceleration (but lower than that found in the group with critical stenosis) or no acceleration.

**Figure 10:**
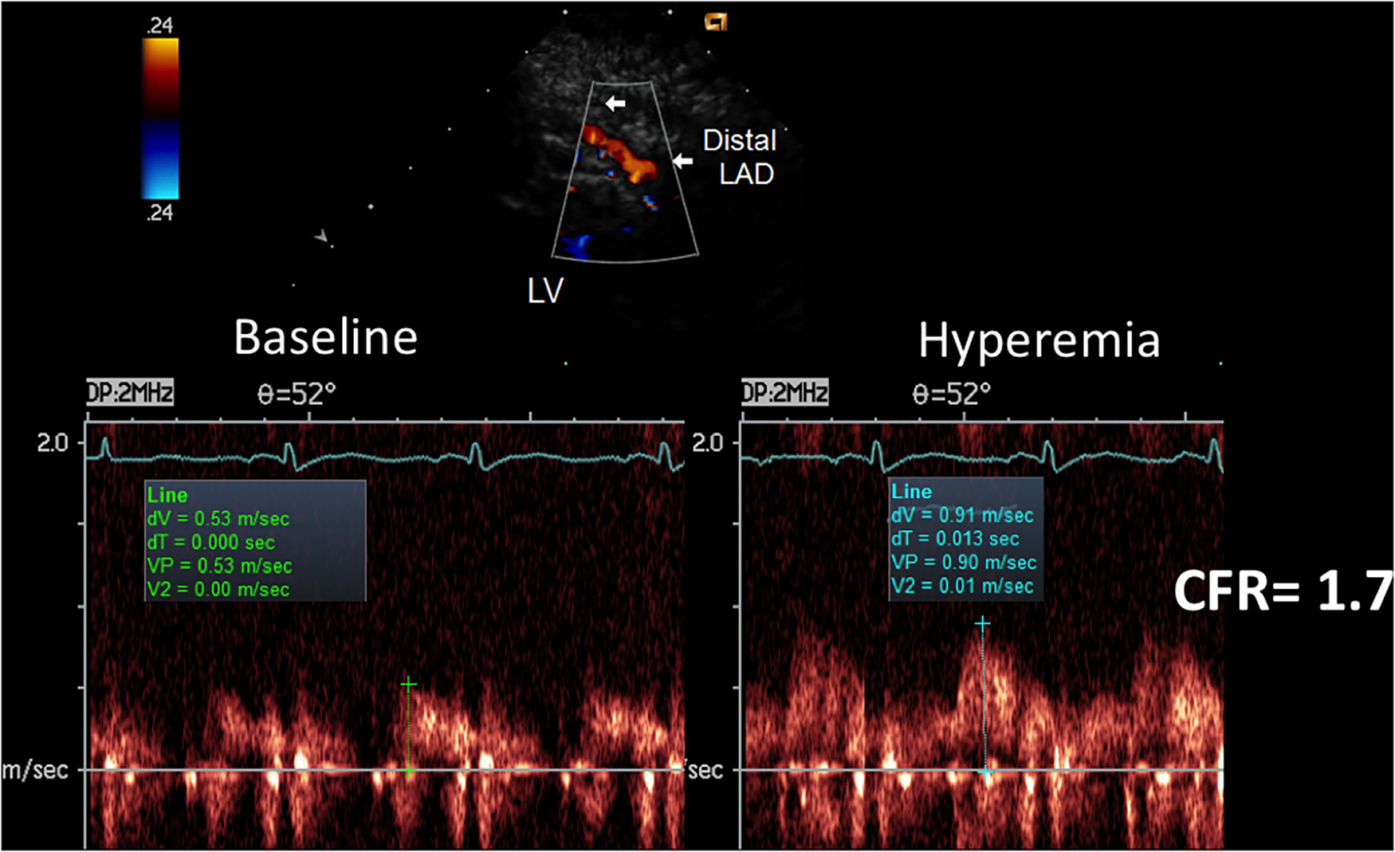
CFR in the distal LAD by E-Doppler TTE in a patient with a subcritical/diffuse LAD athero as assessed by angio. Same layout as the other CFR example. The CFR (peak hyperemic diastolic velocity/peak resting diastolic velocity) is blunted (1.7). The S/D ratio is normal; S/D, ratio of the systolic to diastolic waves of coronary flow velocity.

**Figure 11:**
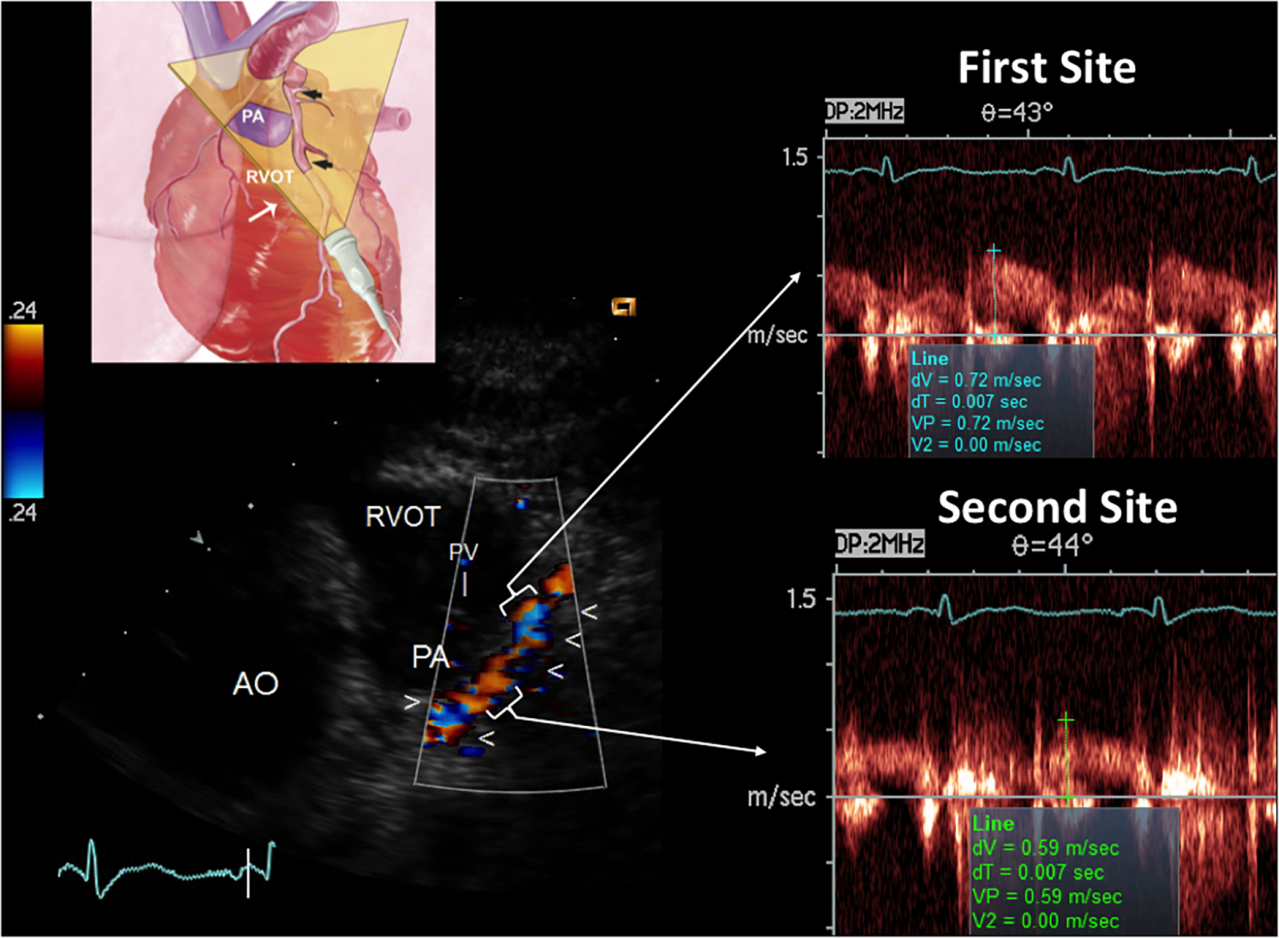
E-Doppler TTE AsF tracking in the LAD in the same patient with the same layout of critical LAD stenosis. The cartoon indicates the plane orientation is different since this is a more caudal approach developed for the mid LAD and the terminal part of the proximal with the same cut. The color flow indicates a dysomogenous color signal with multiple areas of aliasing (encoded in blue) In the right area: pulsed-wave Doppler tracing at the more distal aliasing (upper trace) shows slightly greater BFV than at the reference site (bottom trace) so detecting a mild AsF (22%). By applying the continuity equation (not corrected) that utilizes the VTI (time velocity integral) of the 2 curves (first site and second site) the percentage reduction of the area at the stenosis site is 30% so indicating a mild but apparently diffuse athero as CFR was blunted. In addition, the reference shows high velocity (>50 cm/s), suspicious for diffuse athero. RVOT, right ventricular outflow tract; AO, aorta; PV, pulmonary valve; AsF, accelerated stenotic flow.

**Figure 12:**
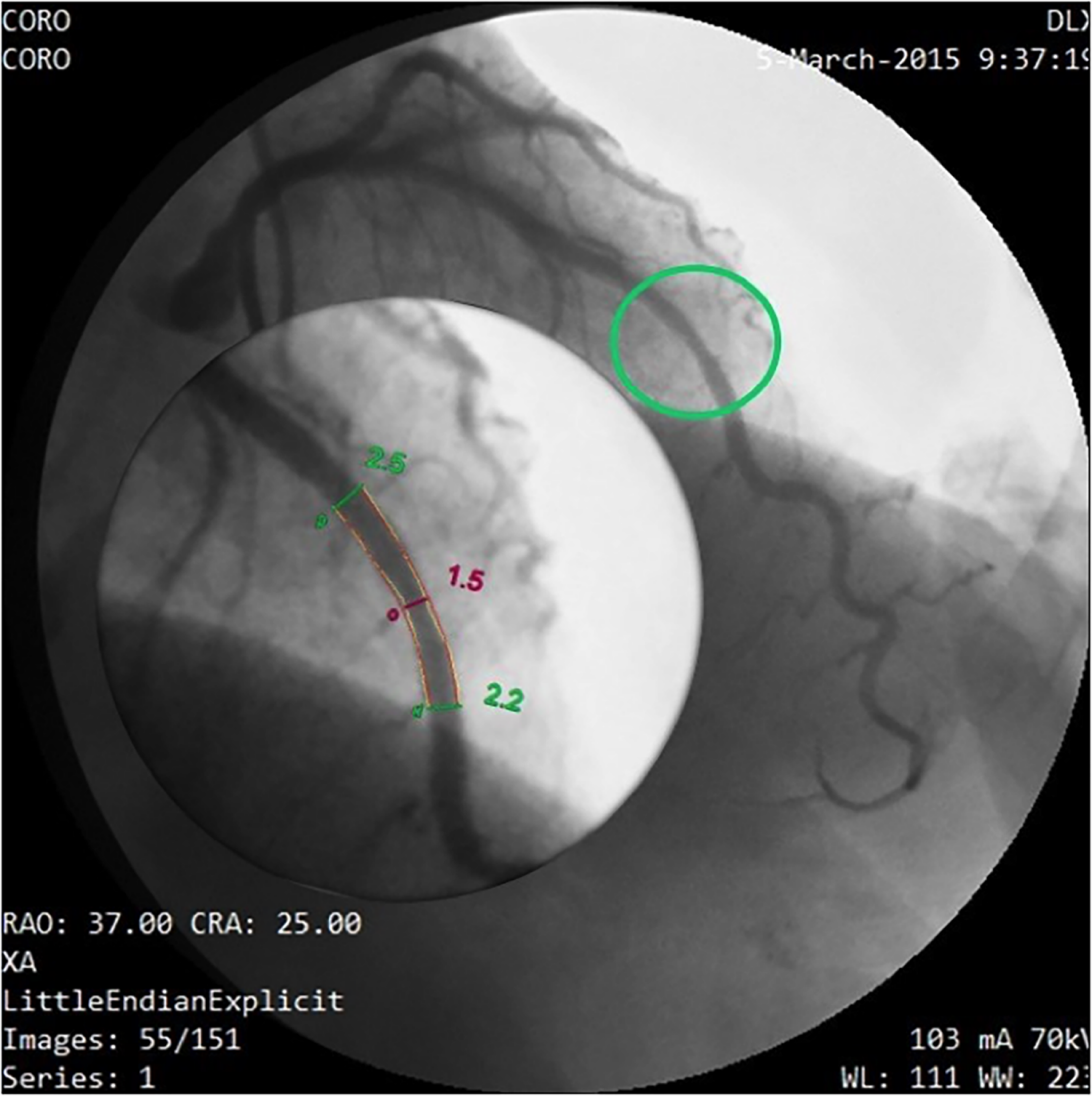
Coronary angiography in the same patient as in Figures 10, 11. Same layout as in the previous angiographic image. In this cranial right oblique view, the site of the mid LAD mild stenosis (encircled in green) is depicted and quantified (40% CSA stenosis) that confirms the E-Doppler TTE results in terms of location ad severity. In addition, angiography shows LAD luminal irregularities and subocclusive stenosis of the right coronary artery (not shown) so clearly pointing out a diffuse atherosclerosis. However, no deduction can be done of the real functional impact of this apparently mild athero. QCA, quantitative coronary angiography; LAD, left anterior descending coronary artery; CSA, cross sectional area.

The presence of AsF was effective in predicting the angiographic signs of subcritical/diffuse athero, as illustrated in Figures 10–12. In particular, first-site BFV in the LAD was higher in the 16 patients with angiographic evidence of LAD subcritical/diffuse disease than in the 11 patients with no angiographic athero (56 ± 16 vs. 41 ± 9 cm/s; mean difference 15 cm/s, 95% CI: 3–26, *p* < 0.01), whereas velocity at the reference sites was similar (41 ± 9 cm/s vs. 40 ± 7 cm/s; *p* = ns). Consequently, the percentage increase was greater in the former (43 ± 32% vs. 2 ± 5%; mean difference = 42%, 95% CI: 24–59, *p* < 0.001), who also more frequently showed at least one localized aliased color signal: 14/16 patients (87%) vs. 1/8 patients (12%) (*p* < 0.001) (see Supplementary Video S2, which shows the movie version of the example reported in Figure 11).

**Figure 13:**
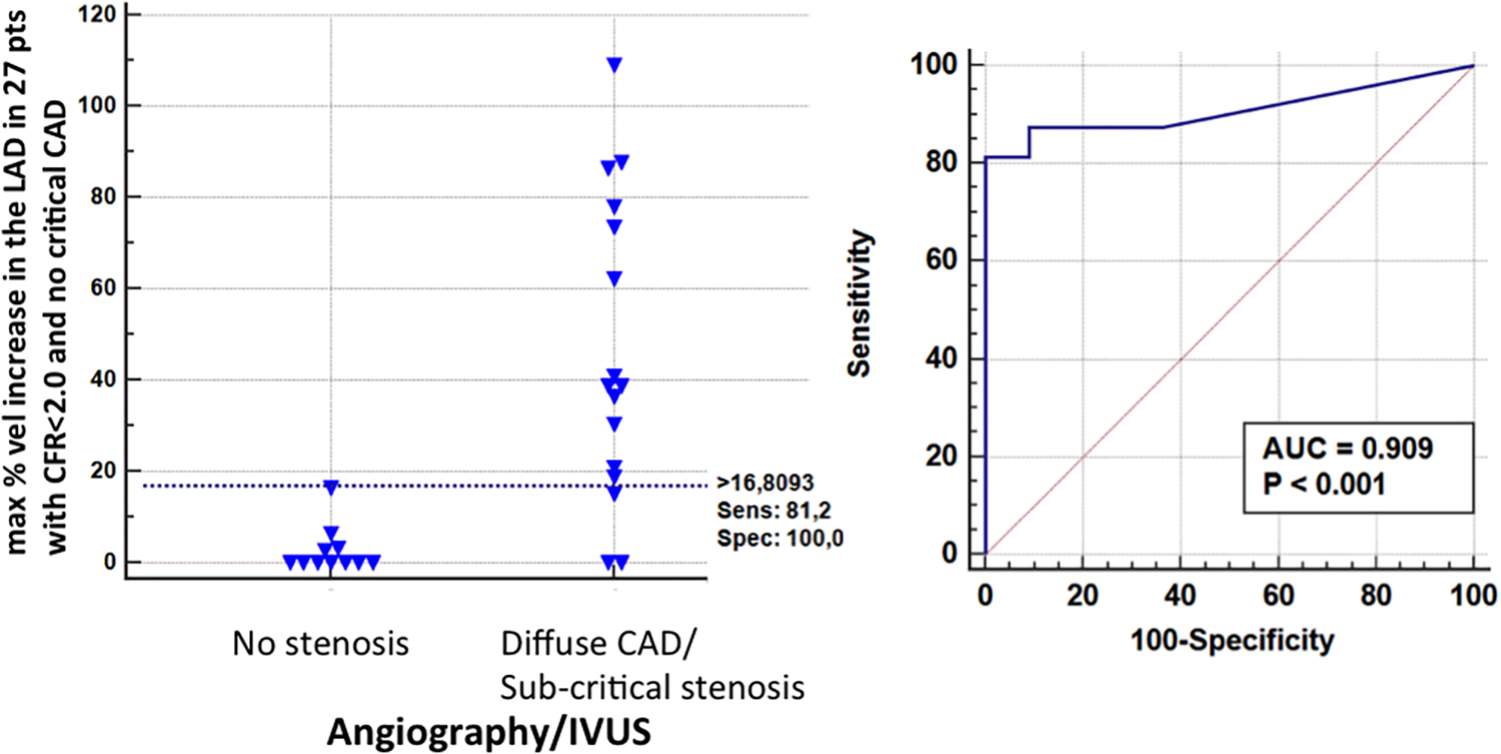
ROC curves in sub-critical/diffuse CAD. Left: individual value bar graph showing the maximum individual percentage increases in velocity in the LAD segments in patients with and without subcritical/diffuse CAD, and the cut-off value (dashed horizontal line). Right: plot of sensitivity against 1-specificity of the percentage increase in velocity (ROC curves) predicting subcritical stenosis by angio in the left anterior descending coronary artery. AUC, area under the curve; vel, velocity; CFR, coronary flow reserve; IVUS, intracoronary ultrasounds.

A BFV % increase using a cut-off value >16% (bootstrapped 95% CI: >6 to >16%), as obtained by ROC curve analysis (area under the curve = 0.91, 95% CI: 0.73–0.98, *p* < 0.001) was able to successfully predict athero (mild/diffuse), as assessed by angio/IVUS in the group without critical stenosis (Figure 13): sensitivity, specificity, positive predictive value, negative predictive and accuracy value were: 81% (95% CI: 54%–96%, 13/16 pts), 100% (95% CI: 71%–100%, 11/11 pts), 100% (14/14 pts), 78% (95% CI: 57%–91%, 11/14 pts), and 89% (95% CI: 71%–97%), respectively.

In 2 patients of the no athero group, high resting uniform velocities in the LAD (≥50 cms), owing to severe hypertrophy secondary to hypertension, and dilated cardiomyopathy with heart failure, explained a borderline blunted CFR value (1.9 and 1.8) (Figure 3) (38).

Thus, pure microcirculatory dysfunction with no epicardial athero was found in only 9 of the 84 patients (10%).

Inter-observer reproducibility. The inter-observer reproducibility was very good; in particular, regarding the repeatability coefficients (37) of the %ASF (% increment of velocity) and % CSA stenosis were respectively 34% (95% CI: 25%–54%) and 19% (95% CI: 13%–29%) (Supplementary Figure 1). Moreover, other variables determined in the inter-observers sub-study showed excellent consistency between the two operators and are reported in detail in Supplementary Table.

## Discussion

Critical accelerated blood flow showed very high sensitivity (95%) and specificity (100%) in predicting critical narrowing, as confirmed by angiography and so explaining a blunted CFR. Most importantly, even a non-critical acceleration was the harbinger of diffuse CAD, as confirmed by angio/IVUS, and explained a blunted CFR, confirming the important energy losses of diffuse albeit not critical CAD. In this report, for the first time, the fluid-dynamic impact of either critical or subcritical but diffuse CAD has been explored with this non-invasive integrated Doppler approach. Finally, by excluding athero based on the absence of accelerated flow (confirmed by angio/IVUS), dominant microcirculatory dysfunction could be inferred as the cause of impaired CFR.

### CFR interpretation by velocity assessment in the whole LAD

The fact that the higher the velocity acceleration the more severe the stenosis and the more blunted the reserve has an extremely solid physical fluid dynamic basis. The most important determinant of stenosis resistance is the minimal cross sectional area of the stenosis, which appears as a second order term in both viscous and separation losses (27). With Doppler mapping of the LAD, the transtenotic velocity is the direct reflection of the minimal lumen area, in other words, the maximal resistance at the stenosis. For this reason, a high transtenotic velocity means greater narrowing at the stenosis site and a higher hyperemic pressure drop, as demonstrated by the inverse relationship between AsF and CFR (Figure 4). This is further confirmed by the optimal prediction of the QCA percentage lumen narrowing by applying the continuity equation, which confirms our previously published data (Figure 9) (22, 23, 39).

The presence of epicardial stenosis reduces the hyperemic flow induced by a microcirculatory vasodilator (adenosine) (40, 41). This is because, at the stenosis, the augmented flow determined by the fall of microcirculatory resistance causes, in a linear fashion, more viscous friction losses and in a quadratic fashion, more expansion losses. Hence the post stenotic pressure declines, as does the flow and finally, the reserve (40–42).

This loss of pressure is entirely correlated with the geometric characteristics of the stenosis.

A real step forward of this method is that it can detect even mild lumen narrowing, as validated with IVUS (23). However, the combination of mild narrowing, as expressed by mild acceleration (<108% velocity increment), with a blunted CFR, strongly indicates a diffuse athero, which blunts reserve. Therefore, a modest acceleration cannot be a benign finding of modest, segmental atherosclerosis if associated with a low CFR (43).

In this series of critical LAD, we found a mildly higher % incremented velocity cutoff (109%) than in a previously reported series (83%) (Figure 8) (22). This is related to the statistically higher rate (20.8%) of stenoses with intermediate severity (50%–70% lumen narrowing) in the previous series than in the recent one (9.1%) (*p* < 0.05). A stenosis visually assessed as an intermediate can easily be hemodynamically insignificant (44). Consequently, the lower cutoff reflects the statistical effort not to consider as false negatives those intermediate stenoses by angio that, in reality, were not functionally critical. The real functionally critical stenosis, therefore, tends to have a higher cutoff than an 83% increment of velocity, as previously reported (22). This was confirmed by a validation study of E-Doppler TTE vs. FFR in which the BFV % increase cutoff that best predicted an FFR <0.8 was a value >123%, very close to that found in this study (109%) (26). These cutoff data were also precisely confirmed by a different group that found a stenotic to prestenotic velocity ratio of ≥2.2 (120% increment of velocity), as the cut-off value for critical stenoses in coronaries by transthoracic Doppler (45).

The low cutoff in the subcritical group (% increment of velocity cutoff >16%) confirms the previous cutoff found for predicting mild athero in comparison with IVUS (>21%) (Figure 13) (23). This indicates that even a minimal acceleration of flow with this method can discern a true mild stenotic plaque that may be isolated or, as in this series, diffuse. Only the CFR can discriminate between these two conditions of mild localized stenosis.

### Feasibility of blood flow Doppler recording in the LAD

The feasibility reported in this study is 100% both in LAD flow mapping and CFR assessment. An enormous step forward has been made in achieving a blood flow Doppler recording in the whole LAD. The enhancement of this echo approach consists firstly of using a very sensitive Doppler technology based on Power Doppler (convergent color Doppler mode), able to detect a low-velocity weak intensity signal such as the coronary flow signal (23). Other sensitive Doppler modules are available on the market (Vivid 7 Doppler technology) (Figure 1). Secondly, the coronary insonification time is prolonged by pharmacologically reducing the heart rate below 60 b/m (23). At this heart rate, the diastolic time is disproportionally lengthened (46) so a longer insonification time is obtained in that part of the cardiac cycle (diastole) where the coronaries are still and clutter artifacts from tissue are absent. Thus, the signal noise ratio is strongly enhanced. This is the main trick to achieve proper blood flow recording.

The technical advances of this enhanced method are new tomographic planes, which add to those first described (22), in particular, the apical approach, which is the only option to tackle the vertically oriented heart, already described for the visualization of the LMCA (29) but recently modified for blood flow Doppler recording in the entire LAD (23); and also the modified low parasternal approach that exploits the left lung cardiac notch (Figure 11). In very difficult cases the use of contrast enhancement must remain the final option, as we had to in a few cases, use the Sonoview® contrast in bolus (12). In this case, the backscatter was best analyzed in the second harmonic using the software available on the Sequoia equipment (12).

### Previous studies

This is the first study that has attempted to combine the CFR with the functional evaluation of epicardial stenosis with the same non-invasive Doppler method. Scanty previous reports in the literature document the potential of flow mapping of the entire LAD epicardial conduit including the LMCA in detecting acceleration and therefore coronary stenosis, but methodologically these reports are limited, hampering the wide applicability of the method, and in any case, the CFR was not measured (13, 45, 47, 48).

Other more recent studies have shown that CFR in the LAD can give added prognostic value to either stress-induced wall motion abnormalities, as assessed by stress echo, or a perfusion defect, as assessed by SPECT studies (49, 50). However, in an individual patient, these combined approaches do not help to exclude an epicardial athero, especially when the disease is diffuse and/or of intermediate severity, where the chance to induce wall motion abnormality is extremely low, even when performing the test at peak stress (51) it does not show the location and the severity of the stenosis and the extension of athero involvement of the epicardial vessel. For this reason, it cannot distinguish microcirculatory from epicardial causes of low CFR. All these limitations combine to diminish even the prognostic power of the combined approach.

### Clinical implications

Our method has obvious, crucially important clinical implications.

The great achievement is that the E-Doppler TTE of coronaries can promptly detect athero and assess its severity in narrowing the lumen thanks to the accelerated stenotic flow, as well as weigh up its hemodynamic impact through CFR evaluation in the distal part of LAD. It can distinguish the athero stenotic involvement of the epicardial conduit (either critical or diffuse) from pure microcirculatory involvement. This has a huge clinical impact on clinical decision-making. Athero involvement with a blunted CFR indicates high risk (43) and must prompt vigorous anti-atherosclerotic reversal therapy (52) not mechanical revascularization therapy (such as transcutaneous transluminal coronary angioplasty) (53). In this respect, this approach is unique, as E-Doppler TTE is an ionizing radiation-free approach that may maximize the effect of reversal treatment. Any ionizing radiation method [Coronary computed tomography (CT) scans in particular], especially if repeated over time, can hamper coronary atherosclerosis reversal treatment. This low radiation exposure, beyond cellular death and DNA damage (54), in conjunction with other risk factors, can cause an acceleration of atherosclerosis by worsening endothelial function (55) and destabilizing plaque (56), meaning it is counterproductive to the reversal treatment itself.

Since the method has high intra-inter observer reproducibility (23) (Supplementary Material), it is ideal for the follow-up in order to assess athero regression-progression. Further studies are needed in this respect.

For the first time, pure microcirculatory disease can be detected with a stand-alone method that has an important impact on clinical decision-making. Although the E-Doppler TTE evaluation is limited to the LMCA/ LAD, the evaluation offers insight into the global CA status since CA is a diffuse process and, in line with autopsy, CT, and IVUS findings (57–59), the LAD is almost always involved in CAD, mainly for hemodynamic reasons (branching system) (60). The absence of a stenotic acceleration in the LAD has a high negative predictive power (96%) for critical stenosis of the left circumflex or right coronary artery (61). Moreover, our highly feasible method can be easily integrated with coronary endothelial function tests (cold pressure test, nitrates) (62) to gain prognostic information that could previously only be obtained invasively (63).

### Limitations

The study has several limitations. In cases of epicardial athero, robustly detected by this method, a certain amount of variable coexisting microcirculatory disease can also be present (Table 1) (64). This is difficult to assess. However, clinical decision-making does not change since in both cases (with or without microcirculatory involvement) anti-atherosclerosis reversal therapy, in general not associated with PTCA (53), is the warranted approach.

Accelerated stenotic flow could also be due to the “ab-extrinsic” compression of the LAD owing to an intra-myocardial course of the mid tract of the vessel. In our experience, this could become an issue only in cases with hypertrophic cardiomyopathy (not present in the reported series). The stiff muscle can compress the vessel also in diastole for a relatively long tract (>2 cm), creating a modest acceleration of the flow for quite a long tract (>1 cm) (65). In other cases, the intramyocardial course is irrelevant, since the compression and the ensuing acceleration are only systolic. In this respect, we believe our approach can be integrated by evaluating LAD wall thickness using high-resolution trans-thoracic echocardiography (66), as the presence of AsF should parallel the increased parietal wall thickness and vice versa. More studies are needed in this regard.

Apart from diffuse athero with prevalent concentric remodeling, uniform high baseline velocity (>50 cm/s) could be an expression of other conditions linked to coronary microvascular vasodilatation in the resting state (functional remodeling). Those conditions that consume coronary flow reserve, apart from anemia and LV hypertrophy more frequently found in patients scheduled for cath, are also heart failure, eventually with high diastolic filling pressure and also systemic inflammation like lupus and rheumatoid arthritis (38, 67). In a general population, a high baseline diastolic flow could be considered as an expression of diffuse athero only when heart failure and systemic inflammation in addition to hypertrophy and anemia have also been excluded.

In this study patients with diffuse disease were relatively few, meaning the data can be considered preliminary, and large confirmatory studies are warranted.

The patients with diffuse CAD had, as a gold standard, angiography and in a few cases IVUS, so a morphological gold standard. A functional approach such as positron emission tomography could confirm, during adenosine, the hemodynamic burden of diffuse subcritical disease by showing signs of subendocardial relatively low perfusion compared to the sub-epicardium (43). However, clinical decision-making is the same, and clinicians would likely pursue anti-atherosclerotic reversal treatment.

## Conclusions

The causes of blunted CFR in the distal LAD, as assessed by E-Doppler TTE, can for the first time be properly discovered with the same Doppler methodology as for CFR assessment, evaluating with high accuracy, the acceleration of blood flow in the whole epicardial conduit. The detection of AsF can reliably identify atherosclerosis lumen narrowing, which would be either critical or subcritical/diffuse (very common). If not present, this likely indicates a pure microcirculatory disease. This new possibility could have a huge clinical impact.

## Data Availability

The raw data supporting the conclusions of this article will be made available by the authors, without undue reservation.

## Supplementary material

SUPPLEMENTARY FIGURE S1

Inter-observers reproducibility of both %AsF and %CSA reduction. That is obtained by plotting the mean (*x*-axis) versus the difference between the two variables (*y*-axis). %AsF bias is −1.38 (95% CI = −11 to 9) and %CSA bias is 2,1 (95% CI = −8 to 3); limits of agreement are reported with 95% CI. The coefficient of repeatability of the two variables is 34% (95% CI 25 to 54) and 19% (95% CI: 14–29), respectively. AsF, accelerated stenotic flow expressed as percent velocity increment at the stenosis site; Diff, difference; CSA, cross sectional area; CI, confidence interval.
