# Supplementary figures and images for: Accelerated stenotic flow in the left anterior descending coronary artery explains the causes of impaired coronary flow reserve: an integrated transthoracic enhanced Doppler study
Source: Front Cardiovasc Med. 2023 Sep 8;10:1186983. doi: 10.3389/fcvm.2023.1186983 (PMC10515222; doi:10.3389/fcvm.2023.1186983)

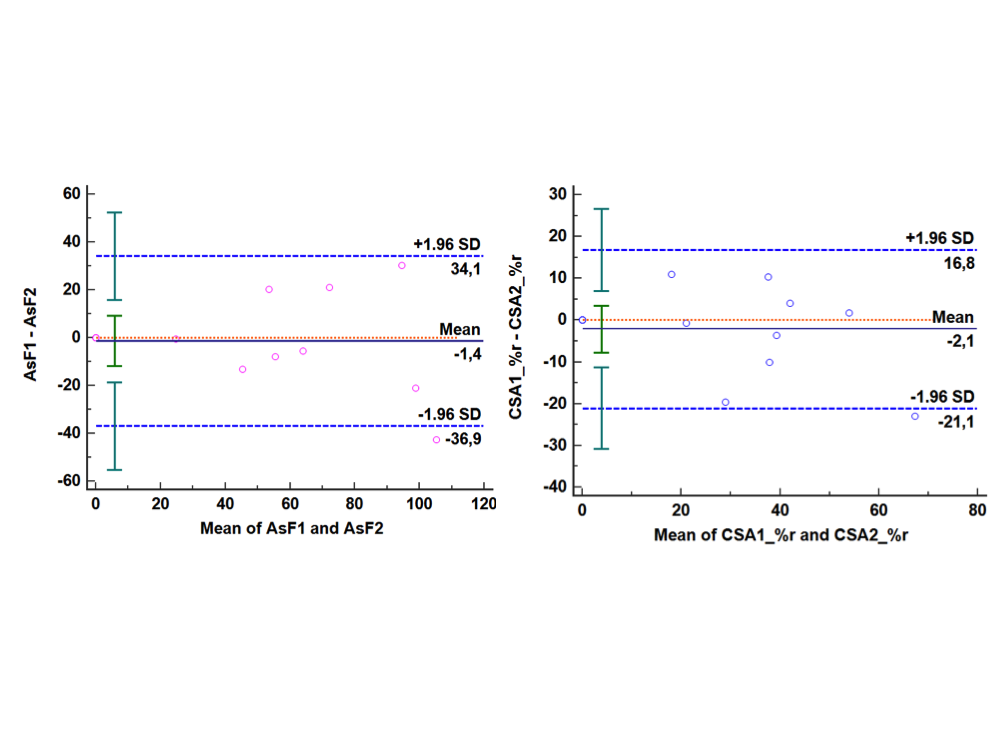

Supplement: Supplementary file 1 [file Image1.tiff]
