# Supplementary material for: Accelerated stenotic flow in the left anterior descending coronary artery explains the causes of impaired coronary flow reserve: an integrated transthoracic enhanced Doppler study
Source: Front Cardiovasc Med. 2023 Sep 8;10:1186983. doi: 10.3389/fcvm.2023.1186983 (PMC10515222; doi:10.3389/fcvm.2023.1186983)
Supplement: Supplementary file 2 [file Table1.docx]

SUPPLEMENTAL METHODS

**Echocardiographic equipment characteristics and settings**. Echocardiography was performed using an Acuson Sequoia™ ultrasound unit (C256 Echocardiography System, Siemens Healthcare, Erlangen, Germany) and broadband transducer (3V2c). The Color Doppler signal was attained in convergent color Doppler mode at 2.5 or 2.0 MHz transmission frequency, while Spectral Doppler was performed in fundamental mode at 2.5 or 2.0 MHz. The color-coded Doppler setting was adjusted to maximize scanning sensitivity (pulse repetition frequency was set at 16 cm/s [2.5 MHz] or 20 cm/s [2.0 MHz] with minor modulation in special cases, and maximizing the sample volume of color flow mapping) without significantly reducing the frame rate (the color box size was reduced to remain in keeping with a frame rate of >30 Hz). All the studies were digitally stored on the built-in dedicated hard drive.

**LAD segmentation and anatomy**. The proximal portion included:^38^ 1) the strictly retropulmonary portion of the vessel extending from the left main coronary artery bifurcation to the plane of the pulmonary valve running horizontally behind the posterior wall of the pulmonary artery (this usually included the first part of the angiographic mid-portion as the first diagonal usually branched off before the pulmonary plane) (Video S1 and Figure 4); 2) the mid-LAD portion that is still spatially oriented as the strictly retropulmonary part running beyond the pulmonary valve plane along the left border of the anterior wall of the right outflow tract (Video S2 and Figure 9).^38^ The distal segment was the strictly vertically-oriented portion of the interventricular segment.

**Parasternal windows (see figures and videos examples in the results section)**. The retropulmonary portion was visualised starting from the second intercostal space. Briefly, after obtaining the short axis view of the aorta, the left coronary fossa was identified as the echo-dense region adjacent to the left coronary sinus, delimited by the left pulmonary artery above and the summit of the left ventricle below.^38^ It was then attempted to visualise the LAD in this area by slightly angling the transducer up and down, and gradually rotating it clockwise in order to deal with the variable inclination of the vessel in the vertical plane (a 0-90° angle).^23^ Once identified in B-mode and then with colour Doppler, the course of the LAD was followed as far as possible (Video S1 and Figure 4).

The approach to the mid-LAD (the upper part of the interventricular portion) has been previously described.^23^ Briefly, the upper mid-LAD was visualised using a lower parasternal short axis view of the base of the heart modified by a slight clockwise rotation of the transducer beam, which allows the transection of the upper interventricular portion of the artery running laterally to the right ventricular outflow tract (RVOT) before it becomes completely vertical (i.e. while it still shares a similar horizontal course to that of the retropulmonary portion) (Video S2 and Figure 9).

**New tomographic plane orientation (see figures and videos examples in the results section).** Visualisation of the proximal and mid-LAD was substantially improved by moving the transducer as far as possible to the left and exploiting the variable extension of the cardiac notch of the left lung and placing the patient in extreme lateral decubitus. The probe was then angled medially and cranially toward the left coronary fossa and the interventricular sulcus: this approach is much better as it eliminates the interference of lung tissue and situates the LAD in the centre of the sector where ultrasound transmission is optimal. A longer tract of the artery (mainly the conjunction of the retropulmonary and the proximal interventricular portion) is insonified on the same plane with a narrow theta angle, and the diagonal branches are also almost regularly insonified (Video S2 and Figure 9). The approach to the vertically oriented interventricular LAD (i.e. the entire distal portion) has also been previously described.^14^

Apical views were also attempted.^24, 31^ Firstly, starting from a 4-chamber view, the probe was angled anteriorly in order to bring the aorta root (oblique slice) into view. A close search for the flow in the left coronary fossa, adjacent to the left side of the aortic root, was attempted by moving the transducer slightly back and forth in order to record blood flow in the LMCA and proximal LAD. Further anterior angling of the transducer allows long-axis insonification of the mid-LAD, which is more vertically oriented and runs along the left border of the right ventricular outflow tract directed slightly toward the right; the transducer was generally moved to an upper intercostal space in order to optimize the mid-LAD window, making it much easier to visualize the blood flow in the diagonal branches.

**Pulsed-wave Doppler mapping Color guided PW Doppler mapping**. With the color guidance, pulsed-wave Doppler mapping in each of the three LAD segments was systematically performed.^20, 23^ If aliasing was found at LAD color flow mapping, we firstly sampled the portion where the color Doppler signal appeared aliased (first site); otherwise, we sampled the whole of the visualized segment in order to obtain the fastest velocity possible. Secondly (second site), we recorded what became the reference blood flow velocity in that coronary segment, which was obtained by sampling proximally or distally to the area with the highest recorded velocity, making sure that the color did not seem to be disturbed. The angle was always corrected and almost always the same at the first and second site (Figure 4 and Figure 9).

E-Doppler TTE scanning is not time-consuming: it takes less than 40 m (range 15 min to 40 min) in most cases (≈70% of cases), while in the most difficult cases (≈30%) and when PW mapping is required for multiple stenosis, the duration is a little longer (up to but very rarely more than 60 min).^24^

**Doppler determination of percentage area stenosis**. As previously described ^23^ according to the continuity equation, blood flow rate should remain constant at the stenosis site (Qs) and in the (proximally or distally) adjacent non-stenotic segment (Qref) provided that there is no branching between the two. As blood flow is derived from the product of the Doppler curve time velocity integral (TVI) and the cross-sectional area of the vessel

 [1]

The percentage area stenosis (% A_s_) can be expressed as

 [2]

and rearranging equations 1 and 2 leads to

 [3]

We also reassessed the data using a corrected formula that takes into account the different flow profiles in the reference and stenotic region: the first parabolic and the second flat.^21, 39, 40^ Doppler measures peak velocity averaged over the cardiac cycle rather than average spatial velocity, but it is the latter (together with the vessel’s cross-sectional area) that is needed to calculate flow. Average spatial velocity is affected by the velocity profile and, as the blood velocity profile in the parabolically shaped reference segment can be expected to be different from that in the stenotic segment, the reference TVI (derived from peak and not average spatial velocity) has to be corrected by the shape factor (0.5) for a parabolic profile.^21, 40^ This avoids overestimating the reference average spatial flow velocity and consequently underestimating %As. Equation [3] therefore becomes

 [4]

**Reproducibility**. Specifically the intra-observer test has been previously assessed by performing the exam twice 3 hours apart by the same operator (CC) in a randomly selected group of 12 pts (13 stenoses, 7 in the proximal and 6 in the mid LAD segment): so the % increment of velocity and % CSA stenosis were twice determined. The inter-observer test has been also previously assessed by performing the exam twice 3 days apart by two operators (CC and PP) in a randomly selected group of 10 consecutive pts (7 stenosis, 4 in the proximal, 2 in the mid and 1 in the distal LAD ): the % increment of velocity and % CSA stenosis were twice determined. In addition LAD global color length, number of LAD segments with aliased color signal, LAD site of each aliased color signal, theta angle correction before color guided PW Doppler recording and duration of examination were also attained twice.^24^

**Coronary angiography and IVUS.** All of the pts underwent coronary angiography using the trans-femoral or trans-radial route depending on the physician’s judgement, and the patient’s anatomy and clinical condition.

All angiographic studies were performed and interpreted in a blind manner since they were performed as routine studies. The coronary stenosis was visually assessed on the basis of multiple projections by one investigator who was unaware of the TTE Doppler results. The presence of minimal luminal irregularities was specifically looked for.

*IVUS*. After reversing any artery spasm by administering intracoronary nitroglycerine, an IVUS examination of the LAD was proposed. The exclusion criteria included anatomic situations in which luminal diameter and/or the site and extent of vascular disease precluded the introduction of the IVUS catheter (i.e. very tight stenosis, diffuse atherosclerosis [in which a normal or near-normal segment of artery could not be identified adjacent to a diseased segment], major tortuousness or significant myocardial bridges) in order to avoid the dissection or destabilisation of the plaque itself, diffuse or isolated coronary spasms, wrinkling or invagination of angled segments by the guidewire, etc.

The total length of the digital IVUS catheter (Eagle Eye Platinum, Volcano Corporation, Rancho Cordova, California, USA) is 150 cm and it has a transverse profile of 3.5F at the transducer. The nominal transducer centre frequency is 20 MHz (free of non-uniform rotational distortion and guidewire artefacts) and it focuses a maximum imaging diameter of 20 mm. It is compatible with 0.014’’ or smaller guidewires, and the probe/wire combination was accommodated in a 6F guide catheter.

After advancing the IVUS catheter under fluoroscopic guidance to the distal segment of the LAD, the probe was withdrawn at a speed of 1 mm/sec using a disposable pullback device (Trak Back II, Volcano Corporation, Rancho Cordova, California, USA) until it came out of the left main coronary artery (LMCA). The procedure was monitored on line using one monitor positioned opposite the operator, and another mounted on the control panel and checked by the US technician. The IVUS data were stored digitally and assessed off-line using dedicated arterial analysis software (Volcano s5iTM Imaging System, Volcano Corporation, Rancho Cordova, California, USA): all of the computer-derived measurements of the digitised images had already been converted into millimetres.

*Analysis of IVUS data*

In accordance with the general angiographic definition, the LAD was divided into three segments: the proximal segment included the first major septal branch or the first diagonal; the middle segment was immediately distal to the origin of first diagonal branch and extended to the point at which the last diagonal branched off. The length of the LMCA, and proximal and mid-LAD was measured using IVUS.^24^

The presence of plaque in each LAD segment was first qualitatively assessed: detecting the atheroma, identifying the tightest stenosis in each segment, and roughly estimating plaque extension (single or multiple) and its possible encroachment into the lumen, ulceration, dissection or intraluminal thrombosis. The main quantitative analysis consisted of the following measurements at each of the most narrowed sites in each LAD segment: first Plaque plus media (or atheroma) CSA (cross sectional area)= EEM (external elastic membrane) CSA minus the lumen CSA; second the Plaque (or atheroma) burden= Plaque plus media CSA divided by the EEM CSA. The atheroma burden is distinct from the luminal area stenosis. The former represents the area within the EEM occupied by atheroma regardless of lumen compromise.^36^
